# Pectoralis minor syndrome – review of pathoanatomy, diagnosis, and management of the primary cause of neurogenic thoracic outlet syndrome

**DOI:** 10.1016/j.xrrt.2022.05.008

**Published:** 2022-06-30

**Authors:** Adil S. Ahmed, Alexander R. Graf, Anthony L. Karzon, Bethany L. Graulich, Anthony C. Egger, Sarah M. Taub, Michael B. Gottschalk, Robert L. Bowers, Eric R. Wagner

**Affiliations:** a Department of Orthopaedic Surgery, Hand & Upper Extremity Surgery, Emory University School of Medicine, Atlanta, GA, USA; b Medical College of Georgia, Augusta, GA, USA; c Department of Orthopaedic Surgery, Sports Medicine, Emory University School of Medicine, Atlanta, GA, USA

**Keywords:** Thoracic outlet syndrome, Neurogenic thoracic outlet syndrome, Pectoralis minor syndrome, Pectoralis minor release, Suprascapular neuropathy, Brachial plexus neurolysis

## Abstract

Thoracic outlet syndrome is an umbrella term for compressive pathologies in the supraclavicular and infraclavicular fossae, with the vast majority being neurogenic in nature. These compressive neuropathies, such as pectoralis minor syndrome, can be challenging problems for both patients and physicians. Robust understanding of thoracic outlet anatomy and scapulothoracic biomechanics are necessary to distinguish neurogenic vs. vascular disorders and properly diagnose affected patients. Repetitive overhead activity, particularly when combined with scapular dyskinesia, leads to pectoralis minor shortening, decreased volume of the retropectoralis minor space, and subsequent brachial plexus compression causing neurogenic thoracic outlet syndrome. Combining a thorough history, physical examination, and diagnostic modalities including ultrasound-guided injections are necessary to arrive at the correct diagnosis. Rigorous attention must be paid to rule out alternate etiologies such as peripheral neuropathies, vascular disorders, cervical radiculopathy, and space-occupying lesions. Initial nonoperative treatment with pectoralis minor stretching, as well as periscapular and postural retraining, is successful in the majority of patients. For patients that fail nonoperative management, surgical release of the pectoralis minor may be performed through a variety of approaches. Both open and arthroscopic pectoralis minor release may be performed safely with effective resolution of neurogenic symptoms. When further indicated by the preoperative workup, this can be combined with suprascapular nerve release and brachial plexus neurolysis for complete infraclavicular thoracic outlet decompression.

Compressive neuropathies are among the most common conditions in the upper extremity.^7^^,^^9^^,^^74^ Compression proximal to the elbow, in the supraclavicular and infraclavicular fossae of the thoracic outlet, is less common. Neurovascular compression in the thoracic outlet is challenging to diagnose and treat.^47^ Thoracic outlet syndrome is categorized as neurogenic (NTOS) or vascular (VTOS), (Table I), with approximately 90%-95% of cases representing neurogenic etiology.^52^^,^^91^ As the brachial plexus and accompanying subclavian vessels traverse the transitioning anatomy of the neck, supraclavicular space, infraclavicular space, axilla, and finally the upper arm (Fig. 1), they are subject to multiple potential compressive sites.^52^ In the supraclavicular area, symptoms occur via narrowing of the scalene triangle and costoclavicular space as contraction of scalenes pulls the first rib superiorly toward the clavicle (Fig. 1, *C* and *D*). Inferior to the clavicle, compression is related to the pectoralis minor muscle (PM) (Fig. 1, *A* and *B*).^91^ Traditionally, brachial plexus symptoms were thought to stem from plexus compression between the anterior and middle scalenes or the clavicle and first rib, termed NTOS. However, recent understanding of the dynamic role of PM in scapular kinematics and nerve compression led to recognition of pectoralis minor syndrome (PMS) as the dominant etiology underlying NTOS.^103^ PMS presents diagnostic and treatment challenges for several reasons. The path of the brachial plexus through the supraclavicular and infraclavicular regions represents an anatomic watershed overlapping between treating sub-specialties. It has traditionally been a gray area between upper extremity, vascular, and neurosurgeons due to the anatomy and varying skill sets regarding nerve surgery, open exploration, and less-invasive arthroscopic approaches.^24^^,^^35^^,^^54^ Due to dearth of strong evidence or consistent diagnostic algorithms, patients with NTOS caused by PMS present with vague symptoms and are often shuffled between primary care, sports medicine, rheumatology, chiropractic, and pain clinics.^102^ This review provides a comprehensive overview of PMS, highlighting the anatomy, dynamic pathophysiology, reproducible diagnostic algorithm, and treatment of this underrecognized etiology of NTOS.

**Table I:** Overview of neurogenic versus vascular thoracic outlet syndrome.

	Neurogenic	Vascular
Distribution	90%-95%	5%-10% (venous >> arterial)
Demographics	Predominantly younger females	Predominantly younger, athletic males
Primary anatomic site	Retropectoralis minor space	Costoclavicular space, scalene triangle
Pathoanatomy	Pectoralis minor hyerpactivity results in shortening and fibrosis Scapula assumes chronically protracted posture, decreasing volume of retropectoralis minor space Compression during arm elevation on brachial plexus cords ± tethering of the suprascapular nerve at the suprascapular notch	Contraction of the anterior and middle scalene muscles superiorly elevates the first rib relative to the clavicle, decreasing costoclavicular space Primarily compresses the subclavian vein and to lesser extent, the subclavian artery Anatomic variations affecting costoclavicular space are common (ex: cervical rib, anomalous scalene, enlarged transverse process, etc.)
Primary Symptoms	Pain about the shoulder, neck, trapezius, and medial scapula, often accompanied by muscle spasms Subjective paresthesias in the arm or hand may be present, but are nonspecific	Hand and finger pain, cold intolerance, claudication, and episodic arm swelling Arm heaviness and easy fatiguability with use Subjective paresthesias in the arm or hand may be present, but are nonspecific
Examination Findings	Tenderness and + Tinel’s over pectoralis minor (and less commonly over scalenes) Scapular dyskinesia Hand atrophy – late presentation (Gilliatt-Sumner Hand)	Unilateral arm swelling and cyanosis Venous distention about the upper arm Raynaud’s-type appearance and skin changes in the fingers
Traditional Maneuvers	Multiple described provocative examinations (Adson, Wright, Roos, Cyriax, etc) are nonspecific, with high false-positive rates even in the normal population	
Measurements (compared to contralateral side)	Pectoralis minor index Medial scapular distance Medial scapular angle Scapular protraction height	
Diagnostic workup	Ultrasound-guided anesthetic injections – target pectoralis minor coracoid insertion ± suprascapular nerve at suprascapular notch ± scalene triangle MR angiogram of the chest – arms down/arms up protocol for dynamic vascular compression MRI of the brachial plexus EMG/NCS of bilateral upper extremities	
Surgical Treatment	Pectoralis minor release (open or arthroscopic) ± Suprascapular nerve release ± Brachial plexus neurolysis	First rib resection (transaxillary or supraclavicular) ± Scalenectomy ± Resection of anomalous anatomy (if present)

*EMG*, electromyography; *MRI*, magnetic resonance imaging; *NCS*, nerve conduction study.

**Figure 1:**
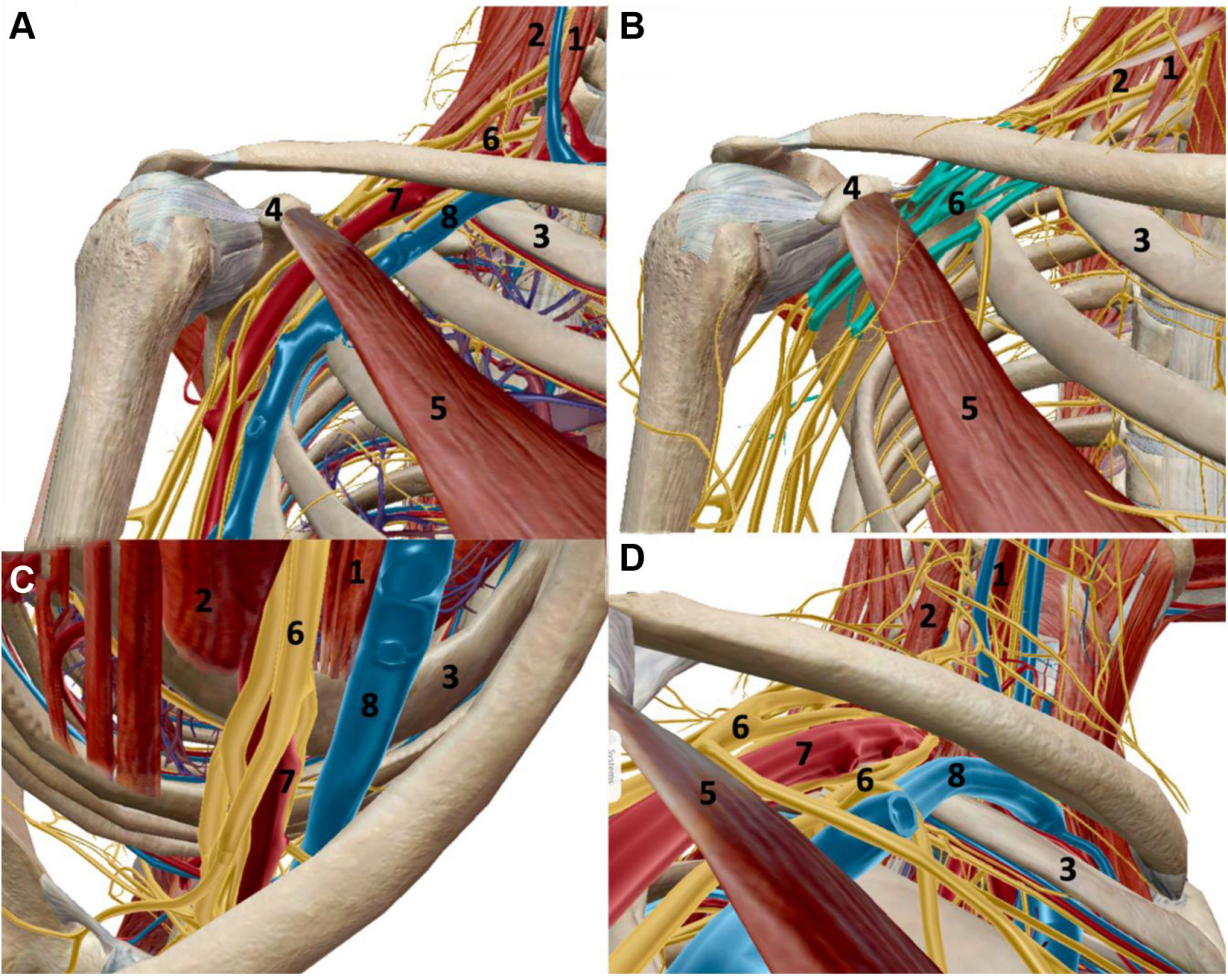
Rendering of the brachial plexus and subclavian/axillary vessels passing through the thoracic outlet in a right shoulder. (**A**) Anterior view with subtraction of the deltoid, pectoralis major, trapezius, rotator cuff, and conjoint tendon. (**B**) Brachial plexus highlighted at the cord level deep to pectoralis minor, with subtraction of the vasculature. (**C**) Inlet view (looking from superior to inferior) of the thoracic outlet. The subclavian vein courses anterior to the anterior scalene in the costoclavicular space. The brachial plexus and subclavian artery pass between the anterior and middle scalenes. (**D**) Outlet view (looking from inferior to superior) of the thoracic outlet. 1, anterior scalene; 2, middle scalene; 3, first rib; 4, coracoid process; 5, pectoralis minor muscle; 6, brachial plexus; 7, subclavian artery; 8, subclavian vein.

## Thoracic outlet anatomy and biomechanics

Sound grasp of thoracic outlet anatomy is imperative to understand potential sites of compression and dynamic contribution of scapulothoracic kinematics that potentiate symptoms. Anatomic understanding further facilitates distinguishing NTOS vs. VTOS (Table I).

**Figure 2:**
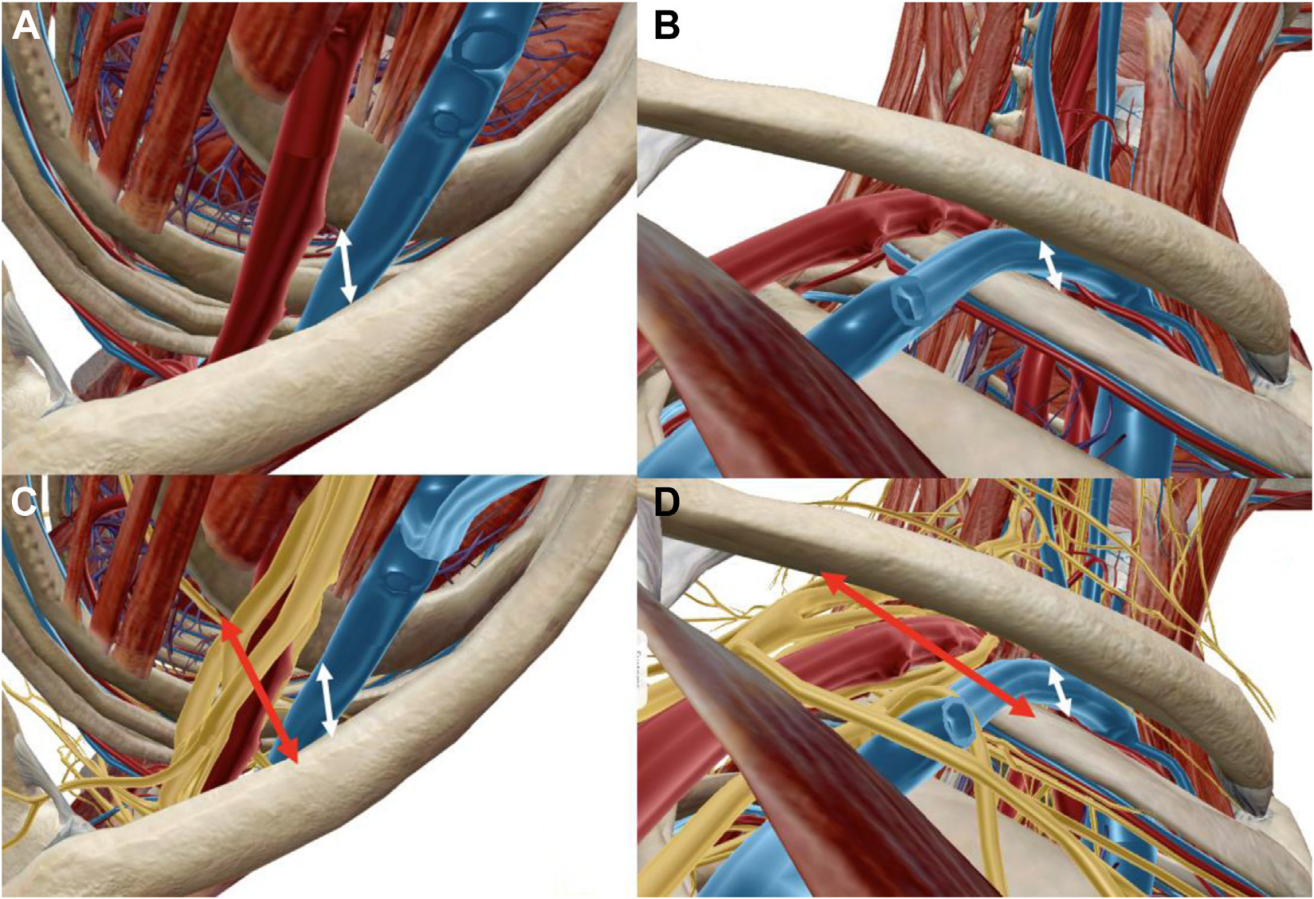
Inlet and outlet renderings illustrating anatomic relationships of the neurovascular bundle and osseous structures. (**A**) Inlet view with subtraction of the brachial plexus, demonstrating proximity of the subclavian vessels between the clavicle and first rib. (**B**) Outlet view depicting similar relationship between the vessels and osseous anatomy. (**C**) Inlet view with the brachial plexus. Note the relatively posterior position of the plexus and increased distance between the clavicle and first rib at this location. (**D**) Outlet view the brachial plexus. Again, note the markedly increased distance between the clavicle and first rib at the posterior location of the plexus.

The thoracic outlet is broadly divided into supraclavicular and infraclavicular fossae (Fig. 1). The supraclavicular fossa contains 2 anatomic spaces: the scalene triangle and costoclavicular space.^26^ The scalene triangle is the most proximal space, bound by the anterior and middle scalenes, and first rib, where the scalenes insert. Brachial plexus roots exit the vertebral foramina and traverse this space, uniting to become the upper (C5, C6), middle (C7), and lower (C8, T1) trunks.^62^ The subclavian artery courses inferiorly within the scalene triangle and anterior to the brachial plexus, in close proximity to the first rib.^29^^,^^83^ Of note, the subclavian vein does not pass through the scalene triangle, instead coursing anterior to the anterior scalene in close proximity to the first rib (Fig. 2).^20^^,^^21^ The scalenes elevate the first rib superiorly and tilt the neck to the ipsilateral side, as they originate from the transverse processes of the cervical vertebrae.^78^ As the first rib elevates, the volume of the scalene triangle shrinks.^39^ The subclavian artery is in the closest proximity to the first rib and is the first structure subject to compression during this dynamic process.^26^^,^^68^ The roots and trunks of the brachial plexus, particularly the upper and middle trunk, are further proximal and posterior, and less likely to be compromised (Fig. 2).^46^^,^^79^ Therefore, compression at the scalene triangle is more likely to create VTOS.

Beyond the scalene triangle, the neurovascular bundle enters the costoclavicular space. This is anterior and inferior relative to the scalene triangle, but due to the curved anatomy of the thoracic wall and clavicle, the long axis of this space is superior-anteromedial to inferior-posterolateral.^32^^,^^48^ The costoclavicular space is bound anteriorly and superiorly by the clavicle and subclavius muscle (originating at the first costal cartilage, inserting on the inferior clavicular surface), medially by the costoclavicular ligament, and posteriorly and inferiorly by the anterior and middle scalene insertions and first rib (Fig. 1).^28^ As the first rib elevates through scalene contraction, the subclavian vein (and lesser extent the subclavian artery) is compressed against the undersurface of the clavicle (Fig. 2).^8^^,^^48^ Simultaneous subclavius muscle contraction or hypertrophy exacerbates this phenomenon.^2^^,^^66^ Variant anatomy at this level, such as cervical ribs or enlarged vertebral transverse processes, preferentially decreases volume in the anterior aspect of the costoclavicular space,^23^^,^^31^ exerting compression on the subclavian vessels. Given the aforementioned orientation and dimensions of the costoclavicular space, the brachial plexus is relatively posterior and less likely to undergo dynamic compression (Fig. 2). Consequently, pathology affecting the costoclavicular space produces VTOS.

**Figure 3:**
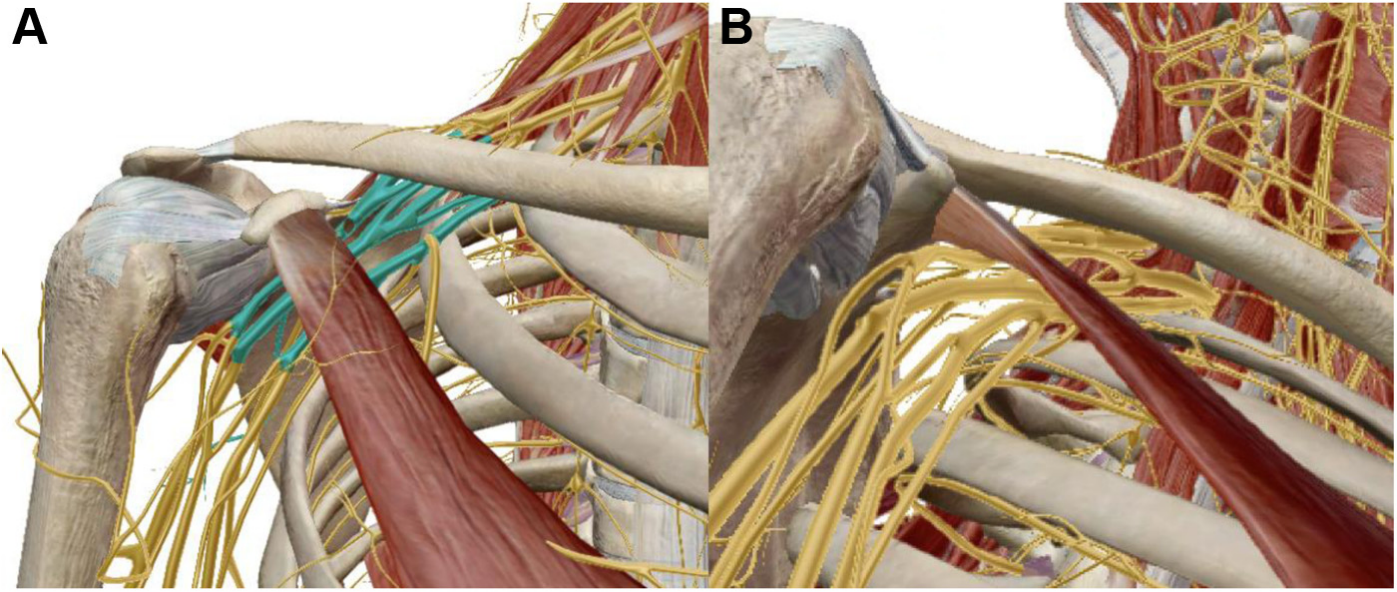
Images illustrating the relationship between the brachial plexus and the pectoralis minor within the retropectoralis minor space. (**A**) Anterior view, with brachial plexus highlighted at the level of the cords. (**B**) Outlet view of the retropectoralis minor space, again demonstrating proximity of the cords to the undersurface of the pectoralis minor.

The neurovascular bundle continues inferolaterally from the supraclavicular to infraclavicular fossa. Brachial plexus trunks split into anterior and posterior divisions, and subclavian vessels become axillary vessels beyond the first rib lateral margin.^58^ The prime space in the infraclavicular thoracic outlet is the retropecotralis minor space (Fig. 1).^42^ This is bound by the coracoid process superiorly, second through fourth ribs posteriorly, and PM anteriorly (Fig. 3).^89^ Within this space, plexus divisions rejoin to form lateral, medial, and posterior cords, and the second stage of the axillary artery continues deep to PM.^100^ The PM is the principal dynamic driver controlling retropecotralis minor space.^89^^,^^91^^,^^94^

**Figure 4:**
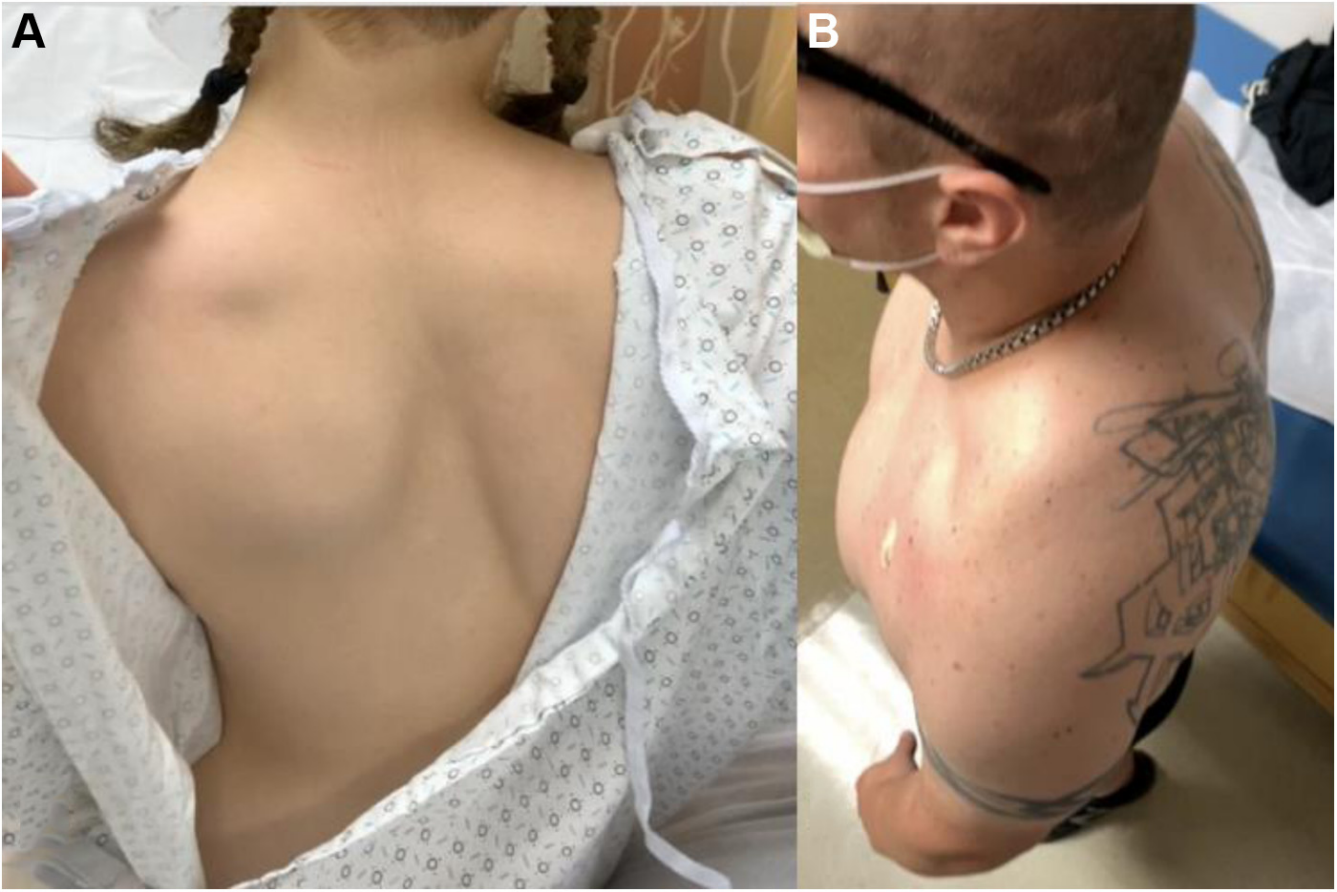
Appearance of scapular protraction in 2 patients. (**A**) Posterior view with protraction of left scapula, creating posterior elevation and prominence of the inferior angle. (**B**) Lateral and superior view demonstrating protracted resting position of the left scapula, with prominence of the inferior angle posteriorly.

The PM originates from the costal cartilage margin of the third through fifth ribs and inserts onto the superomedial aspect of the coracoid, functioning as a dynamic stabilizer of the scapula (Fig. 3).^13^^,^^61^^,^^108^ PM abnormalities cause altered scapular kinematics, particularly during repetitive movements with scapular protraction.^14^ Repetitive movement in forward and downward directions potentiates adaptive changes in the PM in response to scapular dyskinesis.^75^ Over time, hyperactive or spasming PM shortens and develops contracture, leading to protracted resting scapular position (Fig. 4) and altered scapular contribution to shoulder range of motion.^104^ Derangement in dynamic scapular external rotation and abduction alters scapular accommodation to shoulder motion, a well-known feature in various shoulder pathologies.^18^^,^^36^^,^^105^ Patients with shortened PM exhibit scapular dyskinesia manifesting as decreased scapular external rotation/retraction and posterior tilting of the inferior scapula of over 10^o^ compared with controls (Fig. 4).^15^ Patients with scapular dyskinesia often receive incorrect diagnoses of instability, as this pathologic motion pattern is challenging to interpret and diagnose.^95^

Loss of coordinated scapular rotation alters the normal harmony of glenohumeral to scapulothoracic motion, known as scapulohumeral rhythm.^37^^,^^44^^,^^71^^,^^98^ This altered motion, creates chronic compensatory mechanisms, producing impingement from greater tuberosity impaction against the acromion during arm elevation.^49^ This pathologic cascade is especially prevalent and limiting for overhead athletes. McClain et al^69^ noted protracted resting scapular posture in the dominant arms of overhead vs. nonoverhead athletes, attributed to PM shortening. Other kinematic studies of overhead athletes showed similar results, with the dominant arm assuming protracted and anteriorly tilted superior scapular resting position.^80^^,^^85^ Cools et al^27^ further found PM shortening in the dominant arms of elite adolescent tennis players. Evaluating swimmers and volleyball players, Tate et al^99^ and Reeser et al,^84^ respectively, found PM shortening to be a risk factor for symptomatic shoulder pain.

Burkhart et al^19^ coined “SICK” scapula syndrome (scapular malposition, inferior medial border prominence, coracoid pain and malposition, and dyskinesis of scapular movement) as a cause of anterior shoulder pain in overhead athletes. This leads to static anteriorly tilted coracoid malposition with tightening, shortening, and tenderness of the PM.^12^ PM tightening exacerbates scapular malposition, resulting in depression of the anterior acromion and impingement with humeral elevation.^41^ While debate remains whether PM tightness is causative or a consequence of scapular dyskinesis, there is well-established interplay resulting in clinical manifestations of anterior shoulder pain and functional limitation.^57^

The progressive PM tightness, shortening, and fibrosis, combined with anterior coracoid tilt, decreases the volume of the retropectoralis minor space.^42^ As the brachial plexus travels to the axilla and upper arm (Figs. 1 and 3), decreased volume leads to compression of the medial, lateral, and posterior cords and is especially pronounced during overhead activity. Thus, it is by this pathoanatomic cascade that PM tightness creates brachial plexus compression.^90^

**Figure 5:**
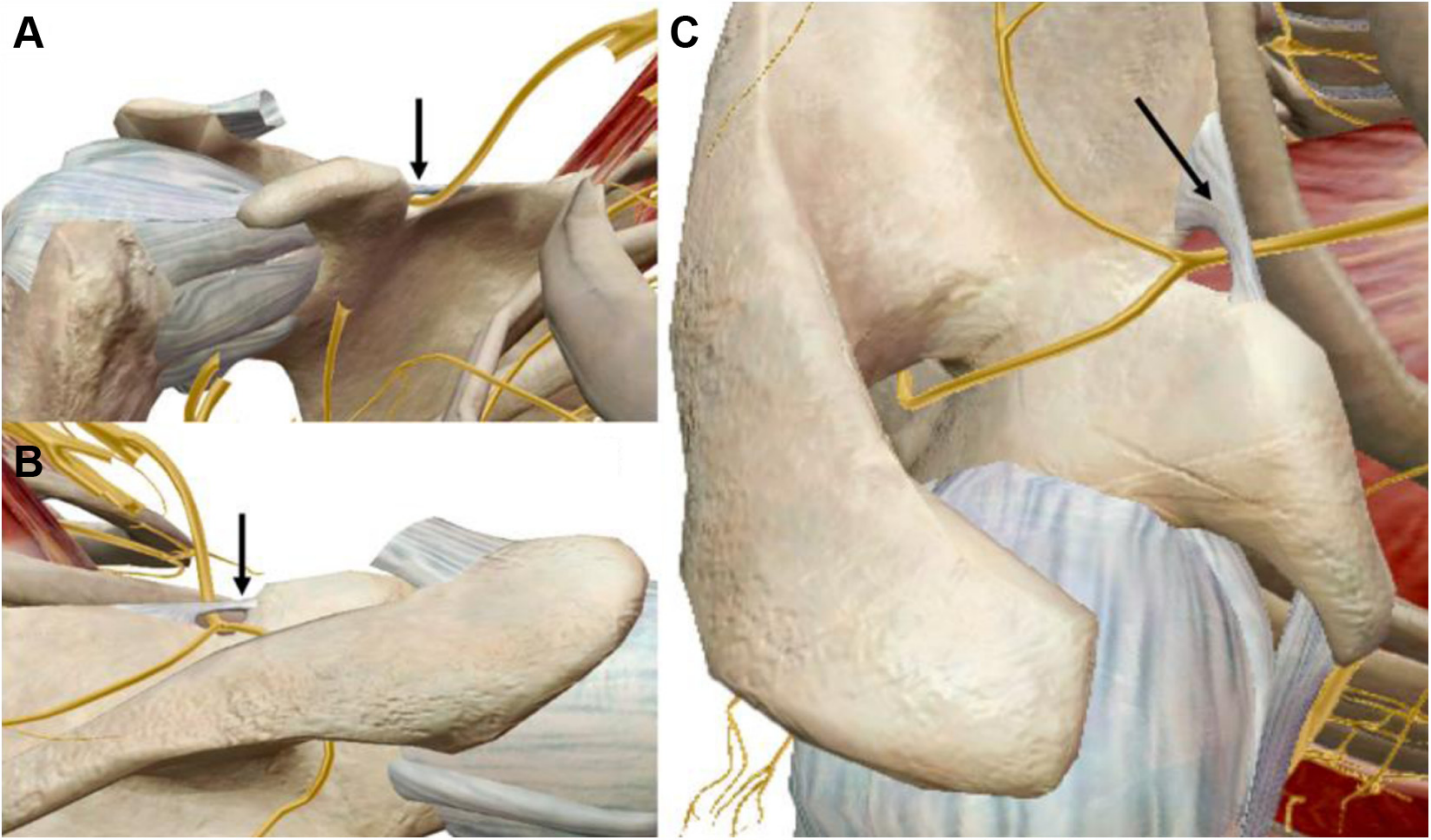
Rendering of the suprascapular nerve and the transverse scapular ligament with subtraction of the deltoid, trapezius, rotator cuff muscles, brachial plexus, vasculature, and pectoralis minor. (**A**) Anterior view. (**B**) Posterior view. (**C**) Superior view. Note how the branch innervating the supraspinatus takes a sharp turn meidally in the supraspinatus fossa immediately beyond the transverse scapular ligament ( indicates transverse scapular ligament).

An additional pathology that simultaneously occurs from this cascade is suprascapular nerve (SSN) entrapment and resultant traction injury at the suprascapular notch (Fig. 5). Chronic protracted scapular posture from PM tightness creates anterior tilt of the superior scapula, pulling the coracoid and suprascapular notch relatively anterior. The SSN is tethered at the suprascapular notch upon entering the supraspinatus fossa due to branching supraspinatus innervation and presence of the overlying transverse scapular ligament.^38^^,^^96^ Therefore, scapular protraction creates chronic stretch injury of the SSN, with symptoms of posterosuperior shoulder pain exacerbated by overhead activity, and even atrophy of the supraspinatus and infraspinatus if left untreated.^53^

## Diagnosis

### Clinical history and physical examination

Diagnosis of PMS is challenging, with patients experiencing chronic symptoms and often undergoing several surgeries, such as open brachial plexus dissection, scalenectomy, and first rib resection, with variable outcomes due to initial misdiagnosis.^42^^,^^45^ Isolated PMS is frequently seen in teenagers or young adults participating in repetitive upper extremity activities.^94^ Sports like baseball, softball, swimming, volleyball, gymnastics, tennis, and weightlifting all rely heavily on scapular protraction and retraction and harmonious contraction of PM. These repetitive scapular protraction/retraction activities lead to irritation and compression of the plexus in the retropectoralis minor space when PM pathology exists.^89^ Young athletes with underlying scapular dyskinesia are at a particular risk for developing PMS.

Physicians must first distinguish whether symptoms stem from NTOS vs. VTOS, which permits identification of site(s) of compression. As detailed previously, robust anatomic understanding of thoracic outlet and dynamic scapulothoracic motion is crucial to diagnosis. In addition to differentiating NTOS vs. VTOS, some authors such as Sanders and Rao^94^ view NTOS and PMS as distinct entities despite similar presentations of anterior shoulder pain, intermittent paresthesia, and weakness, particularly during overhead activities. They further acknowledge significant overlap among the conditions, with approximately 75% of NTOS patients also having PMS, although many have PMS alone.^94^ Based on thoracic outlet anatomy (Figure 1, Figure 2, Figure 3) and scapulothoracic biomechanics, we believe PMS creating compression in the retropectoralis minor space is the prime cause of NTOS. Compression in the supraclavicular thoracic outlet at the scalene triangle and/or costoclavicular space predominantly causes VTOS. Naturally, there are variations or dual sites of compression that can obfuscate diagnosis; however, it is by this fundamental framework of anatomy that we recommend honing one’s differential in patients with suspected thoracic outlet syndrome. Symptoms and signs of PMS are separated into 4 stages (Table II). Stage 1 patients experience vague anterior shoulder pain, primarily during overhead activity. Mild scapular dyskinesia with subtle increased protraction is present and may be missed without detailed attention to scapular motion. Patients tolerate the physical examination, with pain experienced toward extremes of shoulder forward elevation and abduction. Patients typically have not ceased sport participation. Stage 2 presents with worsening symptoms and more severe pain radiating about the shoulder and upper arm. Tenderness over the coracoid is present. Scapular dyskinesia with asymmetric protraction is more pronounced and noticeable on contralateral comparison. Patients either take hiatus from overhead sport or seek counsel during off-season in the hope of ameliorating symptoms for return. Stage 3 presents with hallmark tertiary issues of suprascapular neuropathy and severe scapular dyskinesia limiting function. As PM shortens and the scapula chronically protracts (Fig. 4), constant traction is placed on the SSN in its fixed location at the suprascapular notch. This exacerbates radiating pain about the posterior shoulder, with subjective or even objective weakness during shoulder motion depending on chronicity. Scapular protraction limits shoulder function, as scapular contribution to total arc of motion is diminished. Severe tenderness is present about the coracoid, along with positive Tinel’s sign. Pain and tenderness are present at the medial scapular border, secondary to dyskinesia precipitating scapulothoracic bursitis at the articulation against the chest wall. Patients have completely ceased sports and often sought evaluation with several providers. Stage 4 patients experience severe pain diffusely about the shoulder and periscapular area. There is obvious resting scapular protraction, exacerbated with motion. Severe tenderness and Tinel’s are present over the coracoid, with marked limitation in shoulder function, and atrophy about the infraspinatus. Patients develop compensatory mechanisms to avoid pain exacerbation from the scapular protraction, relying on periscapular stabilizers (trapezius, rhomboids, levator scapulae, and serratus anterior). These muscles contribute to diffuse periscapular pain, adding challenge to the diagnosis. Finally, severe scapular protraction from PM contracture creates chronic anterior stretch of the brachial plexus. From its native relatively posterior position, the plexus is pulled anteriorly in the retropectoralis minor space and throughout the entire thoracic outlet, leading to abutment against the subclavius and anterior scalenes. Resulting tenderness and positive Tinel’s become apparent in the supraclavicular fossa in patients with advanced PMS. Stage 4 presentation can be challenging, as one may examine the patient and confuse this proximal, supraclavicular pain and provocative examination findings as the prime culprit and the distal periscapular and anterior chest pain as secondary effects. However, sound understanding of thoracic outlet anatomy and biomechanics leading to progressive symptomatology ensures correct identification of underlying etiology.

**Table II:** Four stages of pectoralis minor syndrome.

Stage	Symptoms	Clinical signs	Sport/Activity participation
1	Mild anterior shoulder, upper chest, trapezial pain	Subtle scapular dyskinesia and protraction	Able to participate
2	Moderate to severe pain, with additional radiation about the shoulder and upper arm	Localized tenderness ± Tinel’s over coracoid Noticeable scapular dyskinesia compared to contralateral side	Hiatus from sport
3	Severe diffuse shoulder pain Significant posterior radiation as suprascapular nerve involvement worsens Worsening periscapular pain	Severe tenderness and Tinel’s over coracoid Marked scapular dyskinesia with limited arm elevation Tenderness at medial scapula (scapulothoracic bursitis) Pain limited and/or objective weakness of supra/infraspinatus	Completely ceased
4	Stage 3, plus additional pain over supraclavicular fossa and neck	Stage 3, plus additional tenderness and Tinel’s over supraclavicular fossa	Completely ceased

Patients with PMS typically lack positive findings to classic provocative thoracic outlet tests, such as rotational neck maneuvers and Adson, Wright, Roos, and Cyriax tests.^59^^,^^88^^,^^92^ In fact, these maneuvers were found unreliable, demonstrating high false-positive and false-negative rates.^77^ The most precise physical findings for PMS are tenderness and positive Tinel’s over the PM insertion at the superomedial.^6^^,^^90^ Pain and neurologic symptoms are often worsened by the elevated arm stress test, positioning the shoulder in extension and varying positions of abduction to reproduce pain through compression of the brachial plexus between the PM and thoracic wall.^92^

**Figure 6:**
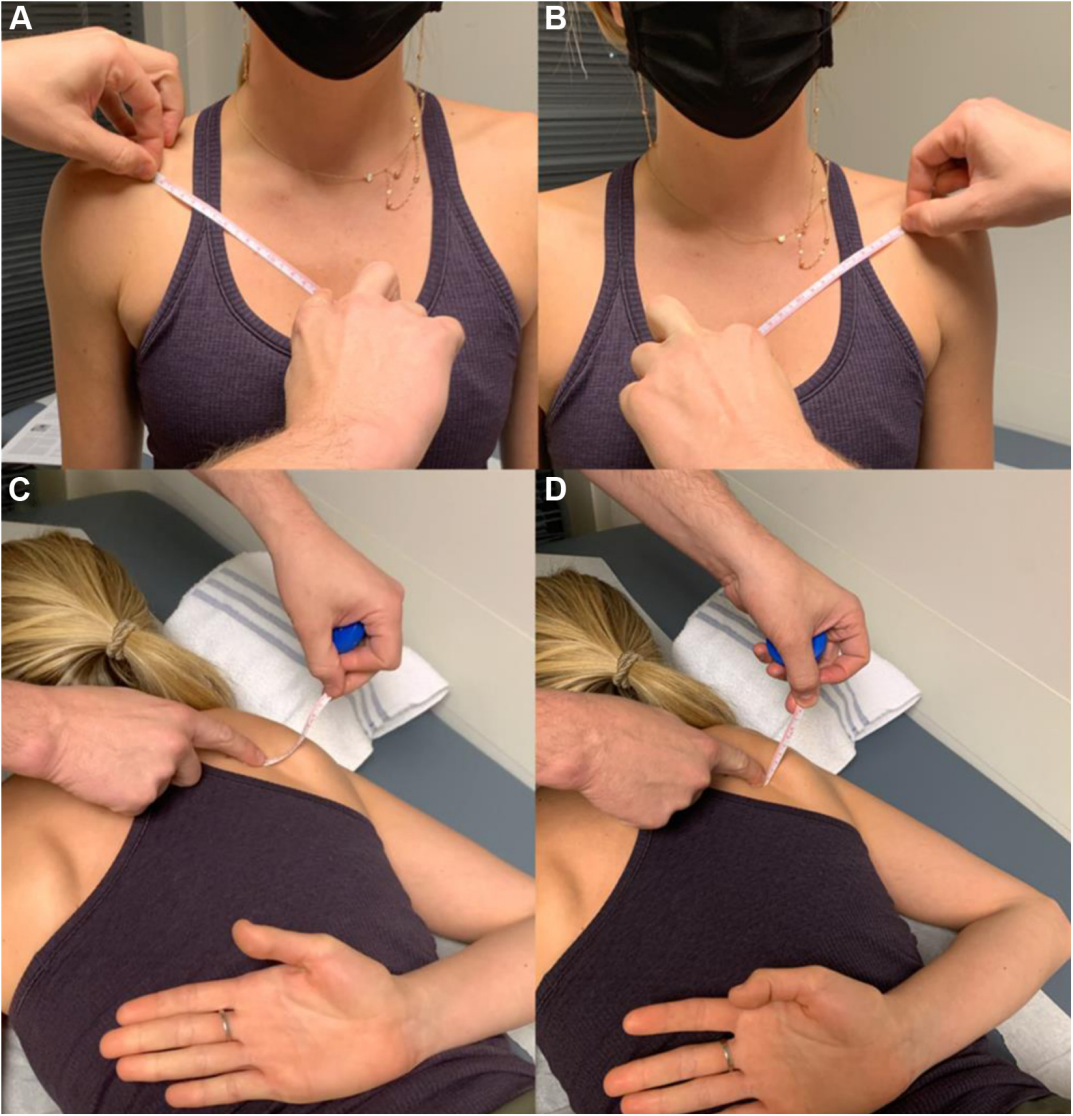
(**A**) Pectoralis minor length and index. Measure the distance from the medial aspect of the coracoid to the inferior margin of the fourth rib at the sternocostal junction with the patient upright. Pectoralis minor muscle index is calculated by dividing this length by the patient’s height (centimeters) and multiplying by 100. (**B**) Measurement of the contralateral side should be performed for comparison. (**C**) Medial scapular distance. This is assessed at the cranial-caudal midpoint of the medial border of the scapula. Dorsum of a patient’s hand is placed on the lumbar spine and a ruler is pressed into the posterior chest wall at the aforementioned midpoint. (**D**) The vertical distance from the posterior chest wall to the medial scapular border is measured. Assessment of the contralateral side should be performed for comparison.

**Figure 7:**
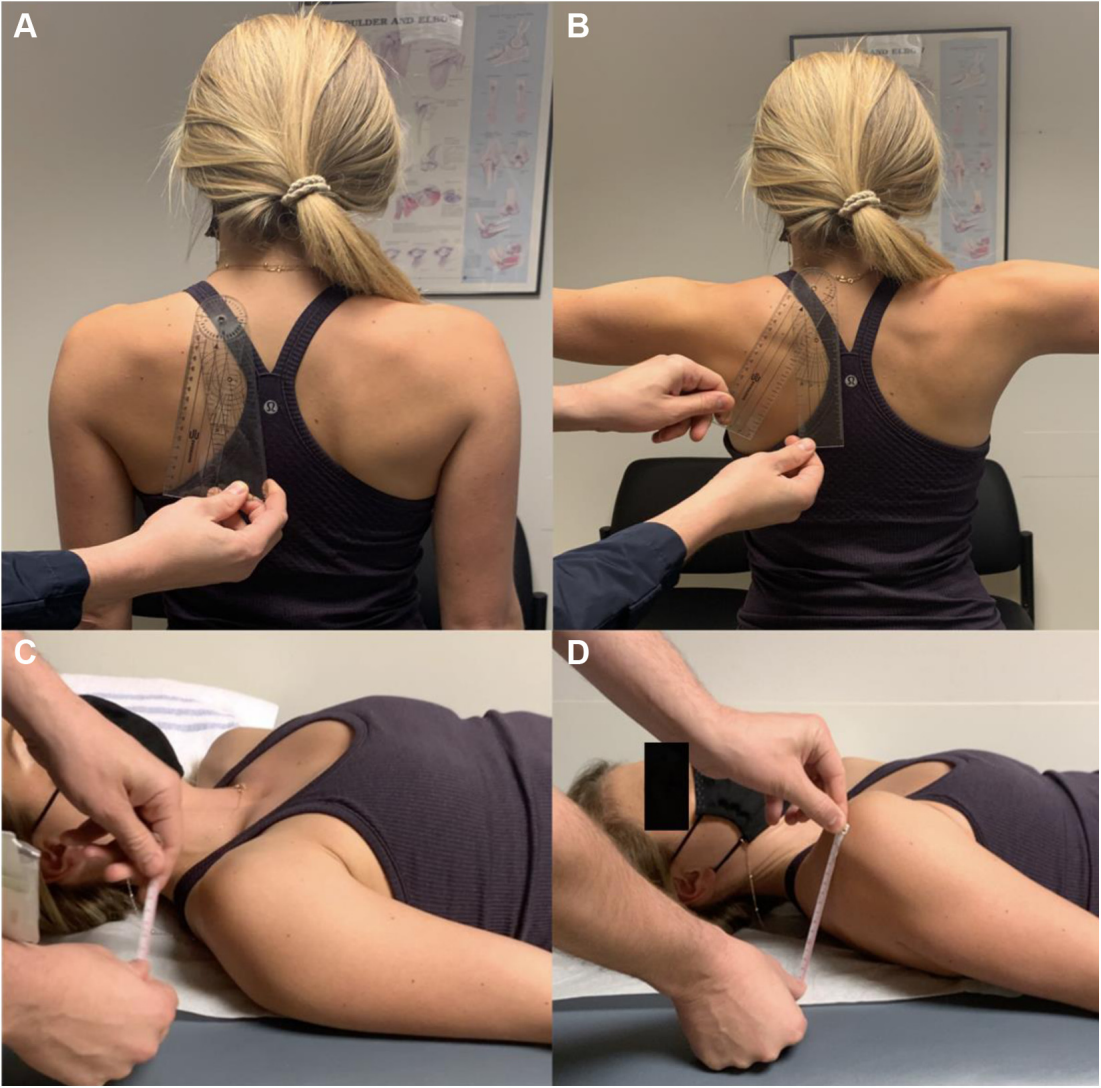
(**A**) Medial scapular angle. With the patient standing upright, first obtain baseline measurement of the angle subtended between a vertical line and the line along the medial scapular border. (**B**) Have the patient abduct their arms to 90° and repeat the measurement. Subtract the baseline angle from the 90° abducted measurement to obtain the medial scapular angle. Similarly assess the contralateral side for comparison. (**C**) Scapular protraction height. With the patient lying supine and relaxed, measure the baseline vertical distance from the table to the posterolateral acromion. (**D**) Ask the patient to actively protract their scapula (bring their shoulder forward without raising their arm off the table) and again measure the vertical distance. The difference between these values is the scapular protraction height. Similarly, assess the contralateral side for comparison.

This is divided by the subject’s height and multiplied by 100 to determine pectoralis minor index (Fig. 6, *A* and *B*). Although simple to measure, there have been challenges establishing normative values and clinically relevant deviations. The medial scapular distance as a measure of scapular protraction is assessed with the patient prone (Fig. 6, *C* and *D*).^82^ Finally, medial scapular angle and scapular protraction height are measured with the patient standing and supine, respectively (Fig. 7). More frequently, PM length and tightness are determined indirectly by assessing scapular position both statically and dynamically, while observing a patient’s scapular motion during simultaneous bilateral arm elevation.^50^^,^^63^^,^^106^

### Imaging

**Figure 8:**
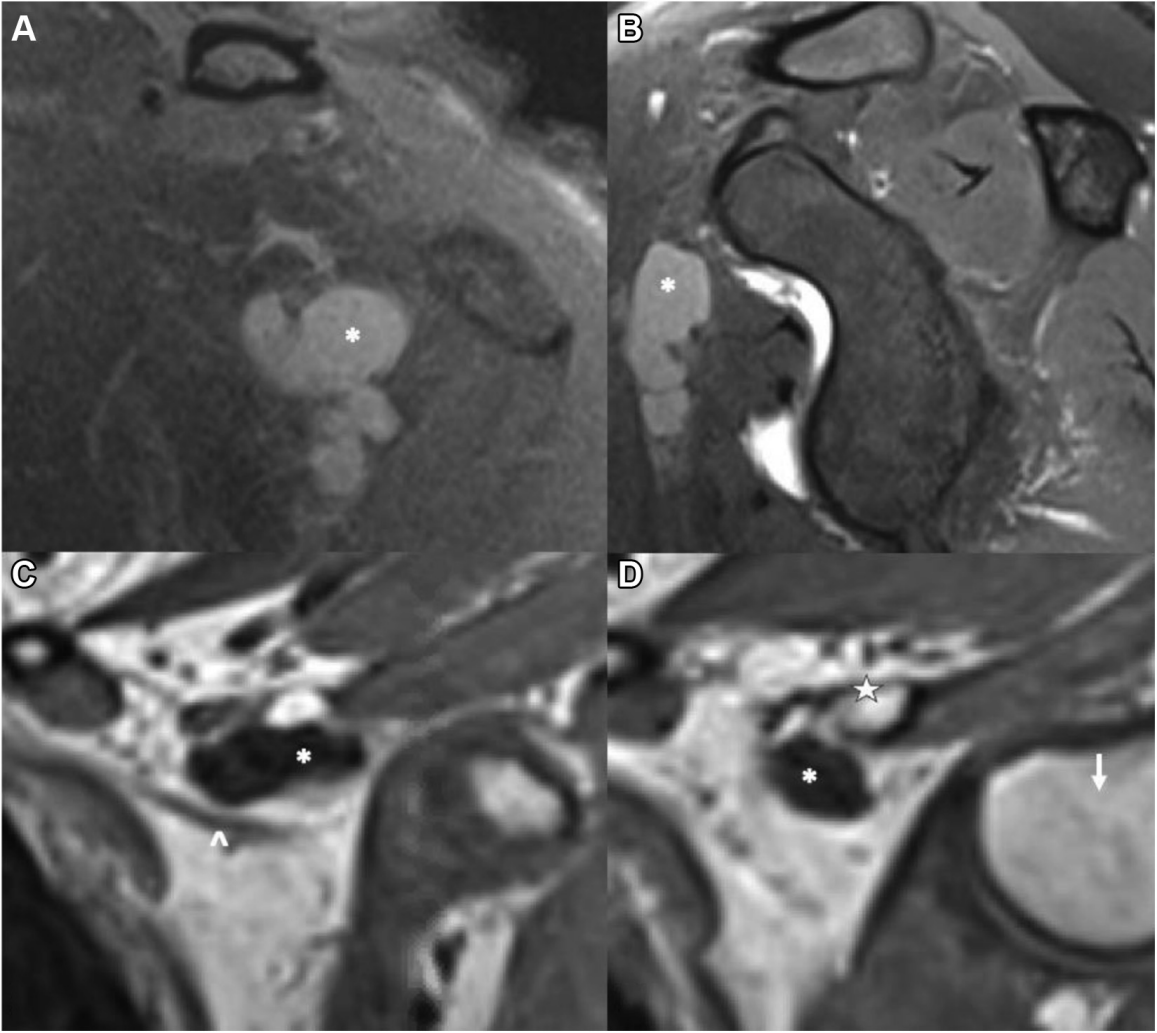
Large subcoracoid cyst noted on MRI of the left shoulder in a patient with vague, deep pain about the anterior shoulder and upper chest, worse with repetitive activity. (**A**) Coronal view, cyst marked with white ∗. (**B**) Sagittal view, cyst marked with white ∗. Dedicated MRI of the brachial plexus was performed, showing proximity of the subcoracoid cyst to the brachial plexus. (**C**) Anterior coronal slice, cyst marked with white ∗, plexus marked with white **ˆ**. (**D**) Posterior coronal slice, cyst marked with white ∗, coracoid marked with white start, humeral head marked with *white downward arrow*. The patient was treated with arthroscopic pectoralis minor release, cyst decompression, and brachial plexus neurolysis, with resolution of her symptoms and return to activity. *MRI*, magnetic resonance imaging.

**Figure 9:**
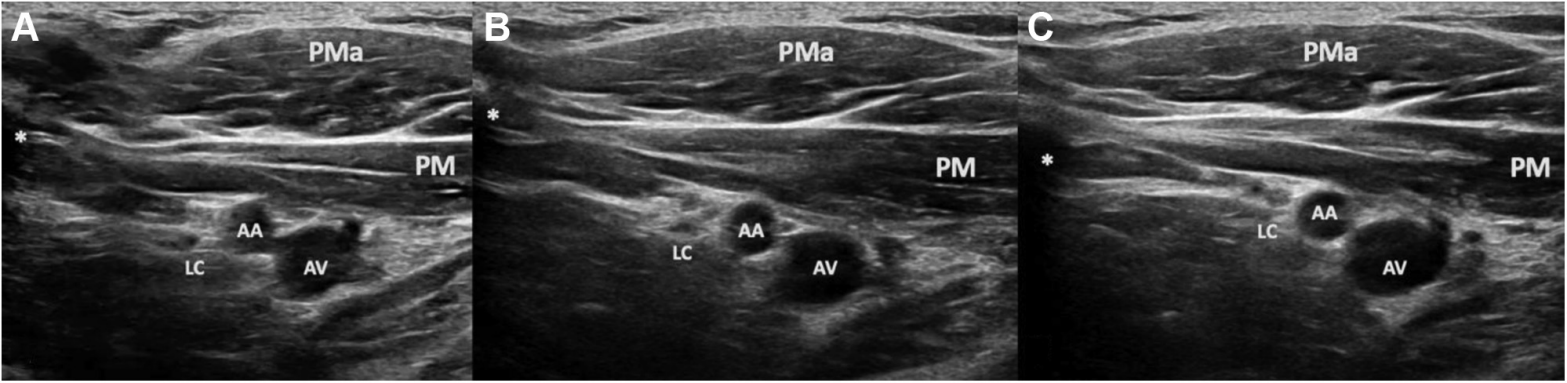
Dynamic ultrasound evaluation of the right pectoralis minor during active arm abduction in the plane of the body, in a normal patient without symptoms. (**A**) Resting adduction with arm at the side. (**B**) 90^o^ active abduction. (**C**) 120^o^ active abduction. Note the absence of posterior pectoralis minor muscle indentation. *AA*, axillary artery; *AV*, axillary vein; *LC*, lateral cord; *PM*, pectoralis minor; *PMa*, pectoralis major; ∗ indicates coracoid.

**Figure 10:**
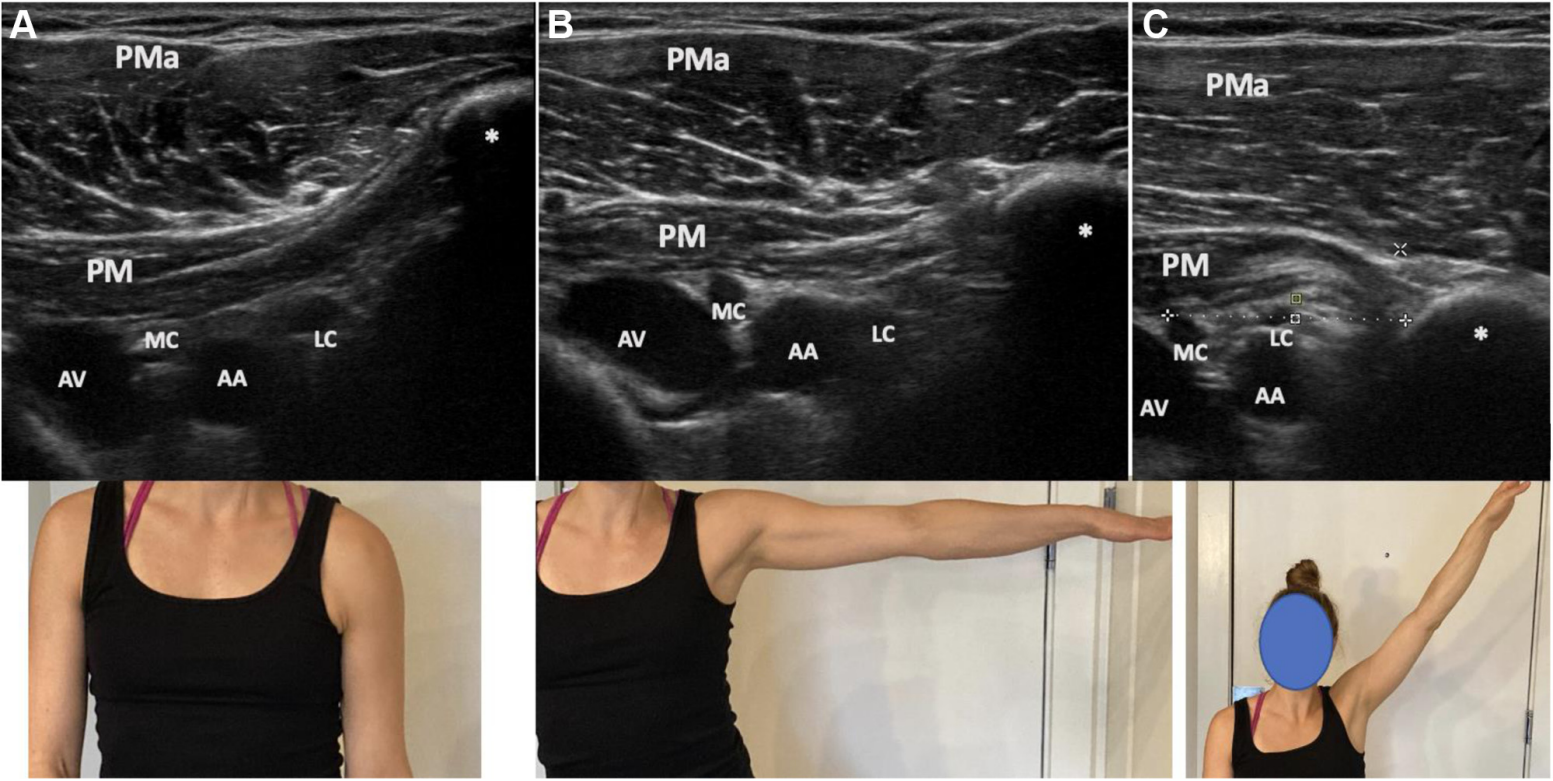
Dynamic ultrasound evaluation of the left pectoralis minor during active arm abduction in the plane of the body, in a patient with symptoms of neurogenic thoracic outlet. Note the indentation of the posterior aspect of the pectoralis minor during progressive abduction, as well as the proximity of the axillary vessels and cords compared to the patient in Figure 6, a consequence of decreased volume in the retropectoralis minor space. Arm at side (**A**), arm in abduction (**B**), and maximal abduction (**C**). *AA*, axillary artery; *AV*, axillary vein; *LC*, lateral cord; *MC*, medial cord; *PM*, pectoralis minor; *PMa*, pectoralis major; ∗ indicates coracoid.

Patients often present with some workup already completed. At minimum, radiographs of the cervical spine and ipsilateral shoulder are obtained. With PMS, these are often normal. However, they rule out the presence of cervical ribs, congenital enlargement of vertebral transverse processes, apical lung masses representing Pancoast tumor, or prior clavicle fracture with nonunion or malunion.^11^^,^^25^^,^^86^ Advanced imaging is frequently utilized, although no specific modality has proven superiority. Magnetic resonance imaging of the brachial plexus evaluates possible sites of compression, nerve edema or fibrosis, or pathology along the plexus mimicking PMS, such as space-occupying lesions or nerve sheath tumors.^110^ The magnetic resonance images are often negative for specific signs of NTOS due to static nature of the test and lack of obvious compressive lesions.^17^ The shoulder magnetic resonance images may demonstrate separate pathologic findings or potential causes for compression, such as subcoracoid cysts (Fig. 8). Neuromuscular ultrasound permits dynamic evaluation of PM during arm abduction in the plane of the body. Compared with unafflicated patients (Fig. 9), those with PMS exhibit posterior indentation of the muscle during arm abduction (Fig. 10), due to shortened and fibrosed PM pressing against the brachial plexus in the shrunken volume of the retropectoralis minor space. Like all ultrasound techniques, there is a user-dependent variability.^97^ Furthermore, no current standard defines normal vs. abnormal with this dynamic evaluation. Vascular workup, including magnetic resonance or computed tomography angiogram, may rule out vascular anomalies prior to surgical treatment. Specific VTOS imaging protocols with arms elevated vs. at the side rule out dynamic elements of vascular.^33^^,^^94^^,^^111^

### Electrophysiologic testing

Electromyography (EMG) and nerve conduction studies (NCSs) are frequently utilized in upper extremity compression syndromes. Nerve compression is quantified as a measured response of latency and amplitude of action potentials. Historically, EMG/NCSs were often normal in the majority of NTOS patients. Recent evidence suggests abnormal NCS response of the medial antebrachial cutaneous nerve may be indicative of PMS, but this is by no means a common finding nor definitive in diagnosis.^3^^,^^67^ One study demonstrated that 40 of 41 patients had at least 1 abnormal finding on EMG/NCSs including latency >2.4ms, latency difference of 0.3 or more between sides, <10 uv amplitudes, and amplitude ratios ≤0.5.^67^ As previously stated, advanced scapular dyskinesia can further cause suprascapular neuropathy via chronical scapular protraction and stretch at the suprascapular notch. The supraspinatus and infraspinatus can be assessed both clinically (atrophy, weakness, or fasciculations noted on physical examination) and via EMG/NCSs.^54^ While these studies confirm the presence of neurologic changes, many patients with NTOS from display negative EMG/NCS results. Ultimately, these tests assist in ruling out alternate compressive neuropathy such as carpal or cubital tunnel syndrome, or cervical radiculopathy, but cannot be relied on alone to diagnose NTOS.^22^^,^^76^

### Diagnostic injections

**Figure 11:**
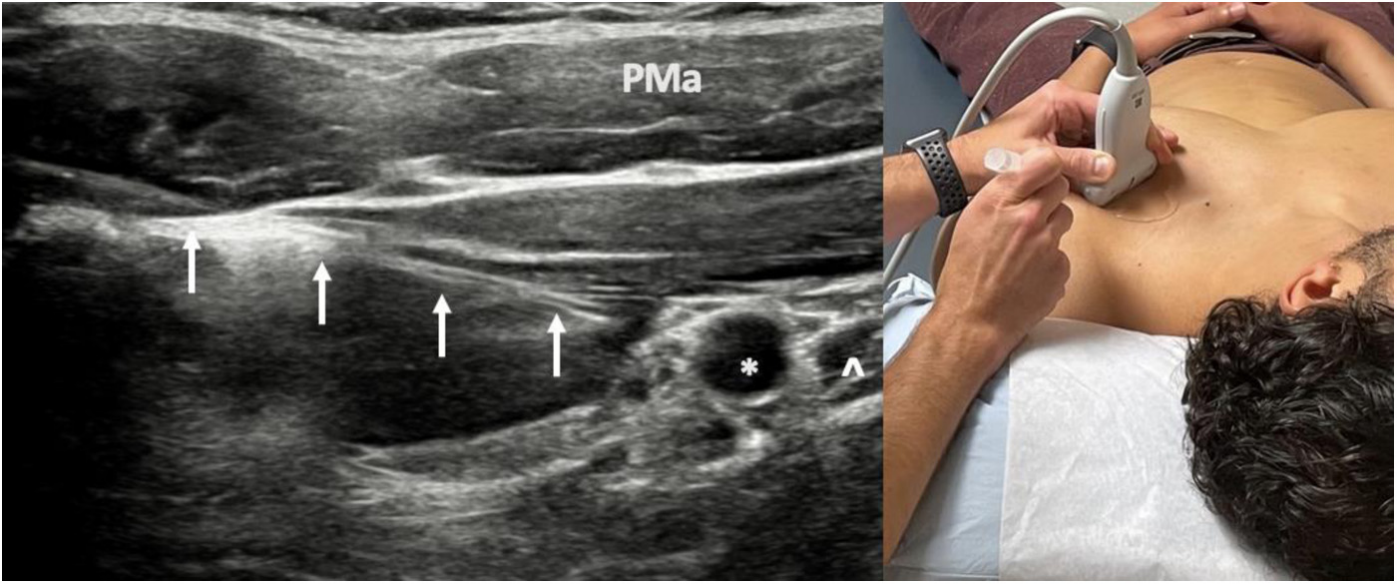
Ultrasound-guided injection of local anesthetic targeted to the pectoralis minor insertion on the coracoid. *PMa*, pectoralis major; ∗, coracoid; **ˆ**, pectoralis minor insertion; *white upward arrows*, needle.

**Figure 12:**
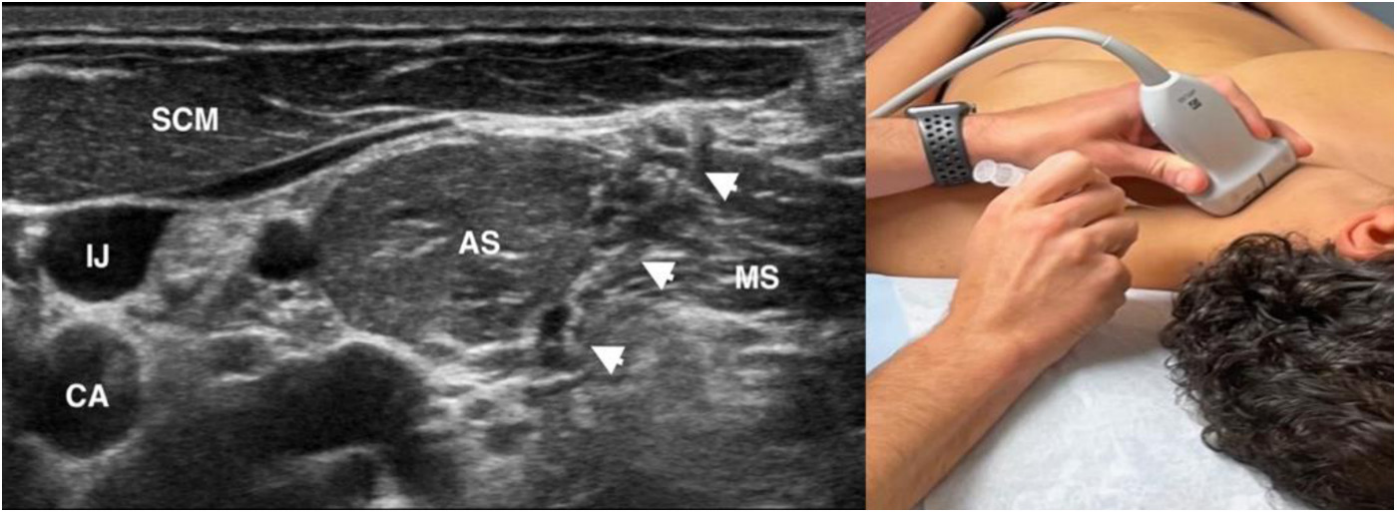
Ultrasound-guided injection of local anesthetic targeted to the scalene triangle. AS, anterior scalene; CA, carotid artery; IJ, internal jugular vein; MS, middle scalene; SCM, sternocleidomastoid; *white arrows*, needle.

**Figure 13:**
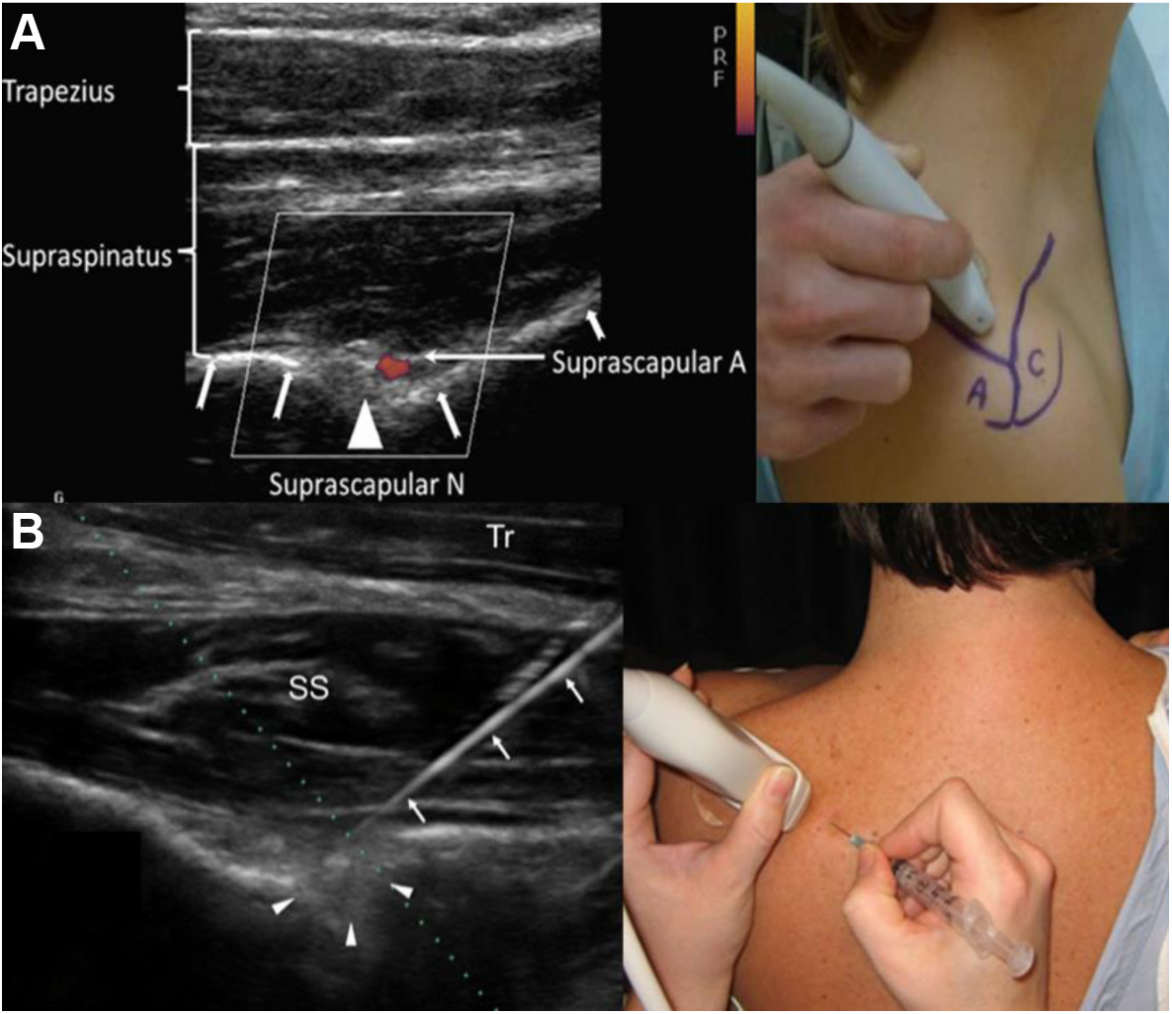
Ultrasound-guided injection of local anesthetic targeted to the suprascapular notch, demonstrating 2 different injection techniques. (**A**) Superior approach, with additional Doppler utilization to identify the suprascapular artery (*white arrows* with split tails demonstrate needle path and infiltration of anesthetic fluid). (**B**) Posterior approach. *SS*, supraspinatus; *Tr*, trapezius; *white arrows*, needle; *white arrowheads*, infiltration of fluid in the suprascapular notch.

Injection of local anesthetic is the gold standard in diagnosing brachial plexus compression syndromes, including PMS. PM injections are performed under ultrasound guidance for accuracy, targeted just deep to PM insertion on the coracoid (Fig. 11). Following injection, the patient is assessed for pain relief,^16^ deemed successful (positive) if >50% relief of pain or paresthesias is achieved. Positive injections are associated with better outcome after surgical treatment.^35^^,^^73^^,^^94^^,^^95^ If symptoms are not improved by injection, or if the patient has symptoms localizing to the supraclavicular area, a separate targeted scalene block (Fig. 12) can assess for potential proximal involvement in advanced stage 4 NTOS.^16^^,^^65^^,^^91^ Guided scalene injections are also correlated with favorable response to surgical intervention.^16^^,^^65^ In patients with concomitant SSN compression secondary to chronic anterior tilt of the superior scapula (Fig. 5), ultrasound-guided injection targets the suprascapular notch (Fig. 13). Botulinum injections targeted to the scalenes or PM are another option, but these are less effective at predicting surgical outcome compared to anesthetic blocks with or without corticosteroids.^65^

## Treatment

### Nonoperative management

**Figure 14:**
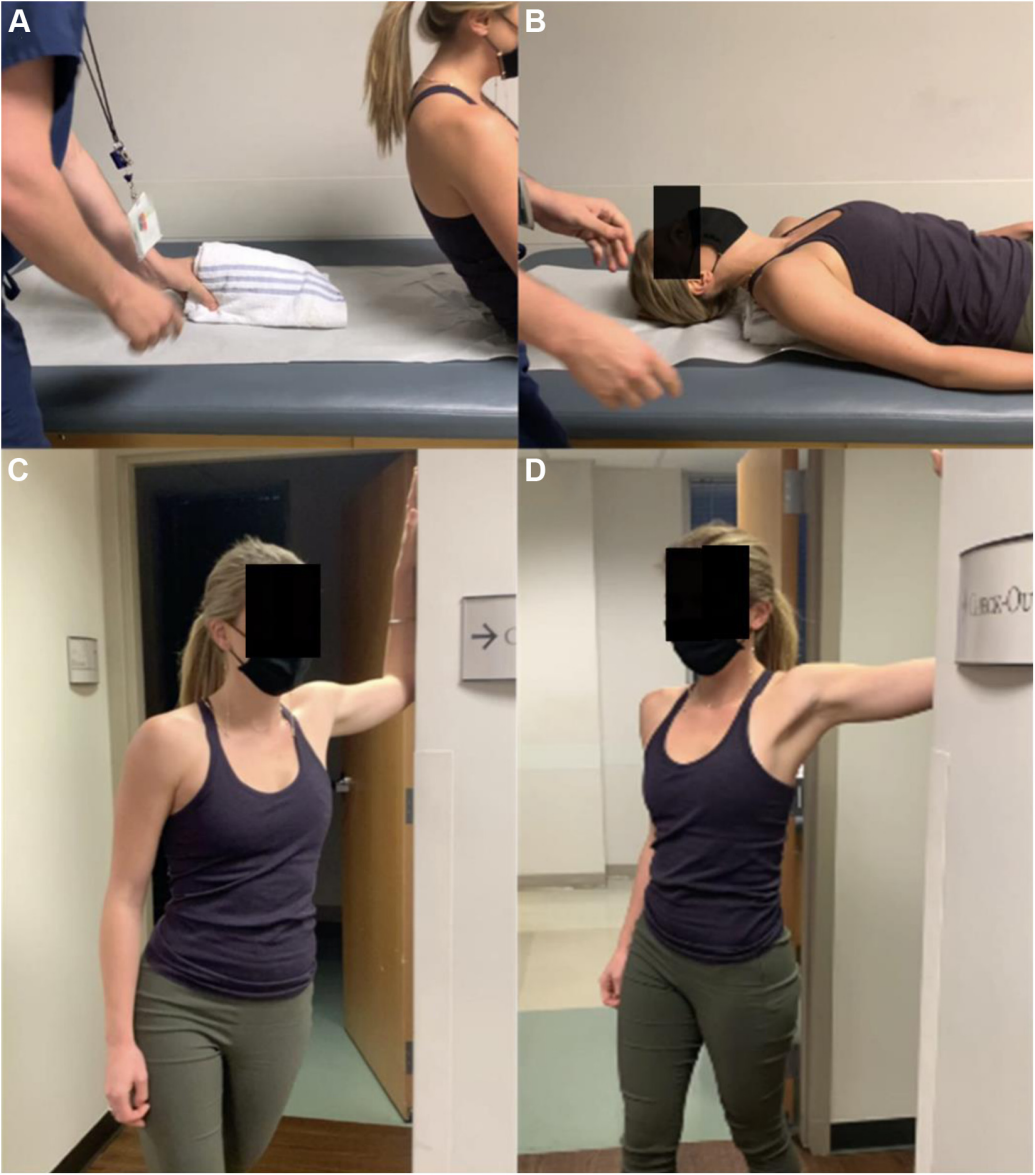
(**A, B**) Center spine towel roll stretch. The patient lays supine with rolled towel between the shoulder blades, allowing the shoulders and scapula to drift posteriorly, stretching the anterior shoulders, chest, and pectoralis muscles. (**C, D**) Corner stretch. The patient stands with contralateral foot forward and arm abducted and externally rotated 90° against the wall. The patient turns their body away from the wall, stretching the pectoralis muscles, anterior shoulder, and chest.

**Figure 15:**
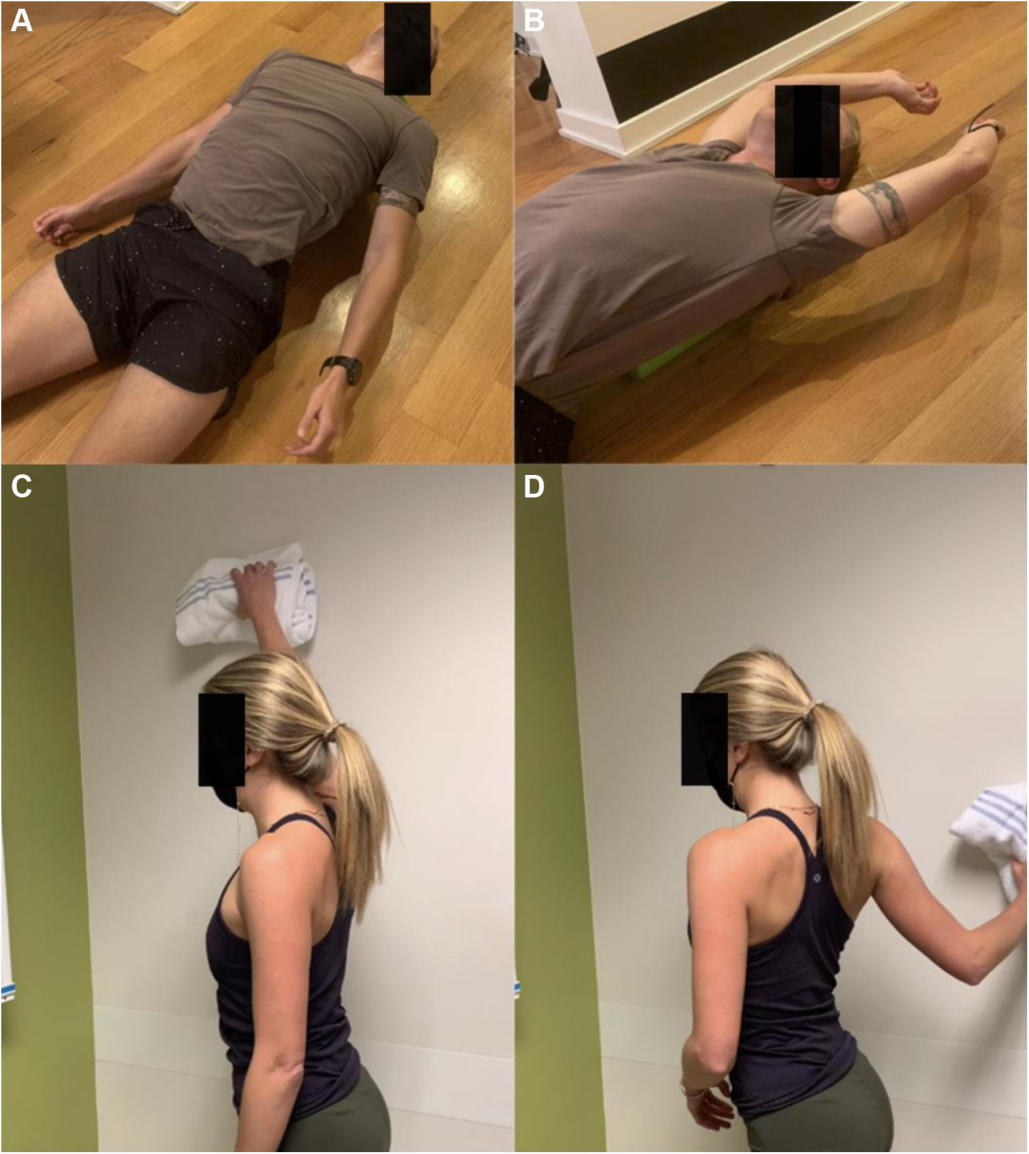
(**A**) Butterfly stretch. The patient lies supine on foam roller or rolled towel between their shoulder blades. (**B**) Stretch begins with arms extended at the side and progresses via abduction of arms to an overhead position in a controlled fashion. (**C, D**) Wall wash. The patient pushes a folded towel against the wall, sliding the towel up and forward with scapula protraction in a diagonal motion, and then reversing the motion downwards and in extension using scapular retraction.

**Figure 16:**
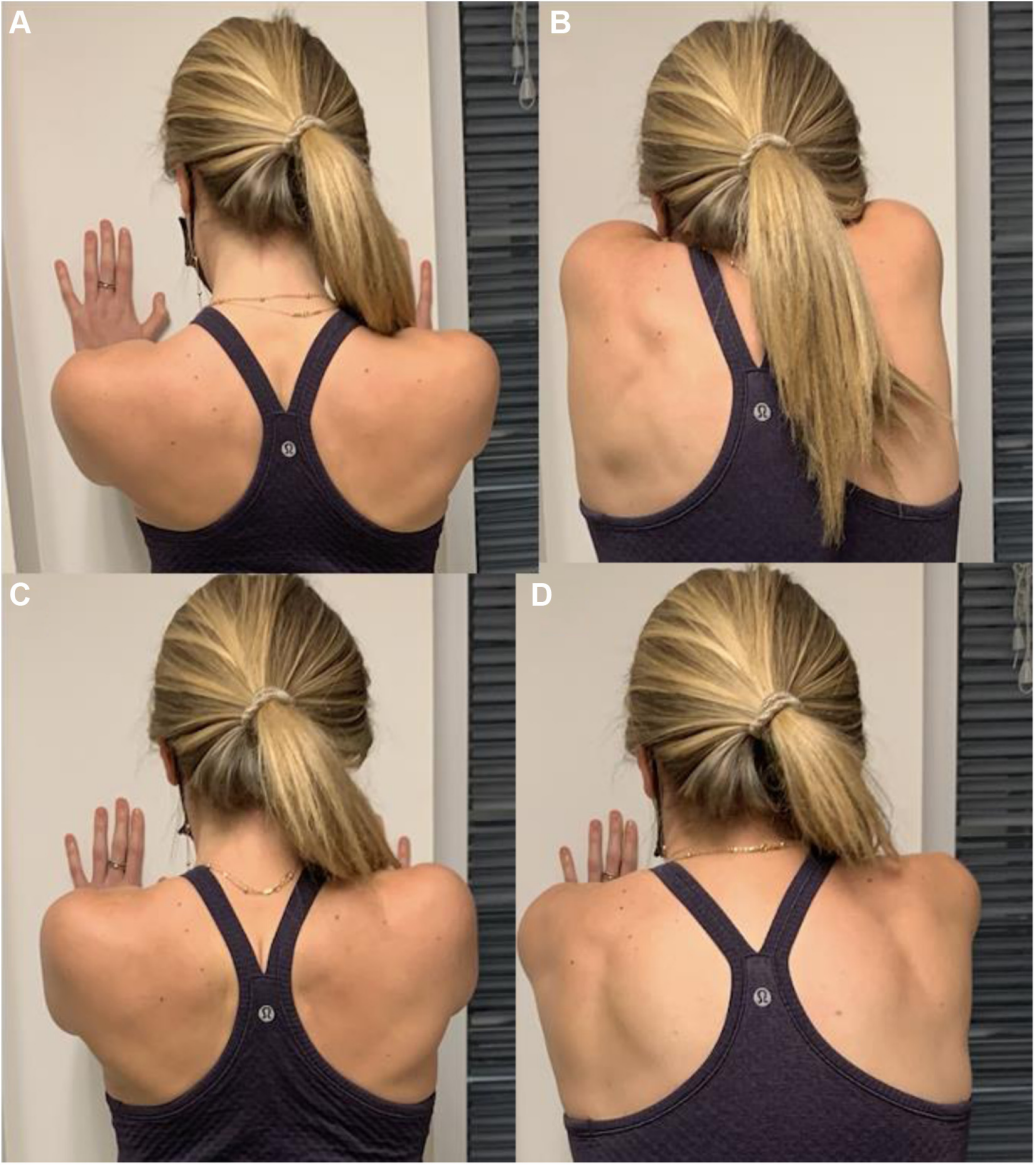
Scapular retraining exercises performed standing upright with arms in forward elevation and hands pressed against a wall. (**A, B**) Scapular depression and elevation. (**C, D**). Scapular retraction and protraction.

**Figure 17:**
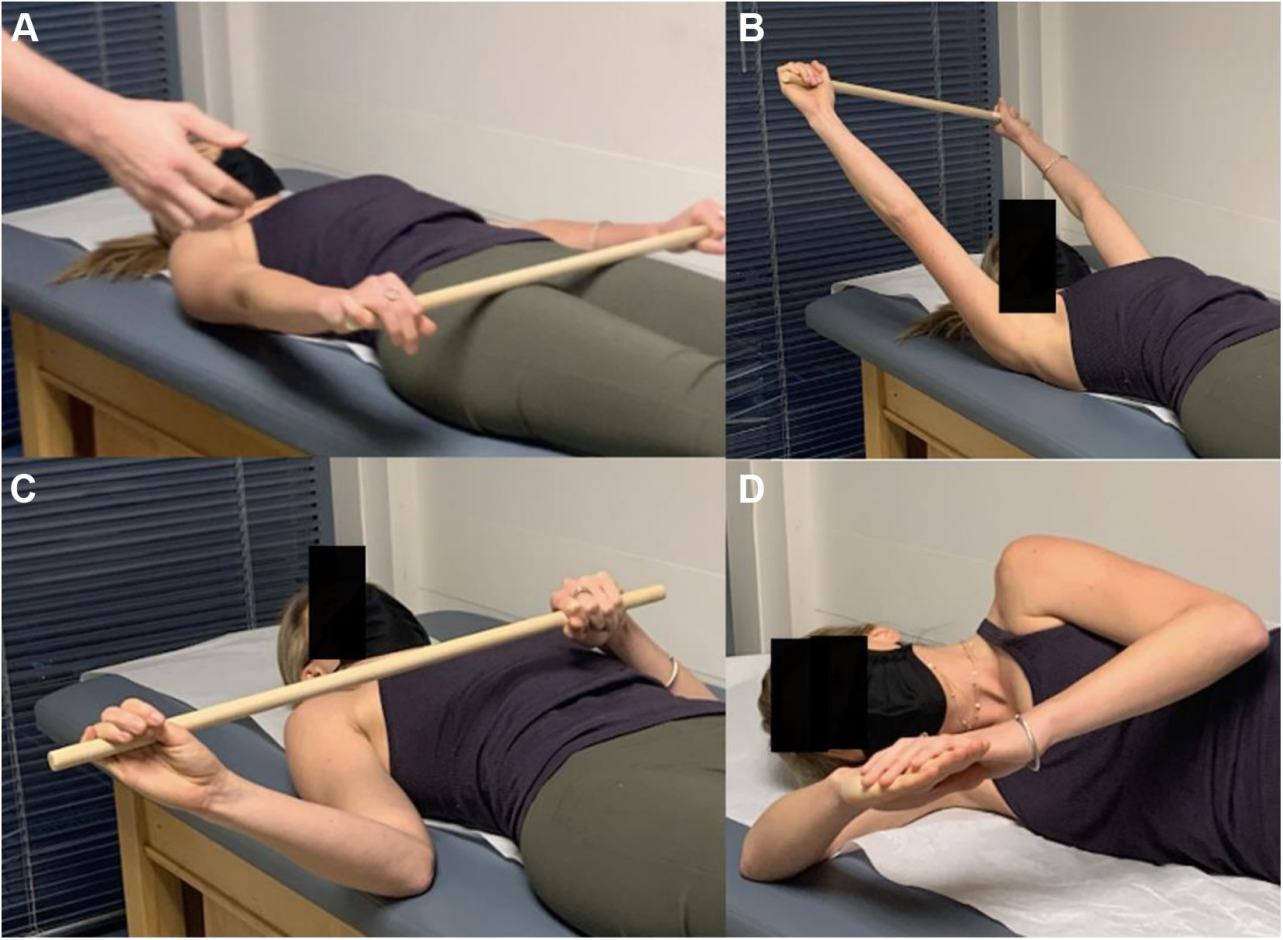
Scapular stretching exercises. (**A, B**) Shoulder flexion stretch, performed supine whilst holding a stick in both hands, permitting the normal arm to assist the compromised arm in attaining maximal overhead flexion stretch. (**C**) External rotation stretch, similarly performed supine to allow contralateral arm to assist with attaining maximal external rotation stretch for the anterior shoulder anatomy. (**D**) Sleeper stretch, performed in lateral decubitus to stretch the posterior shoulder anatomy.

**Figure 18:**
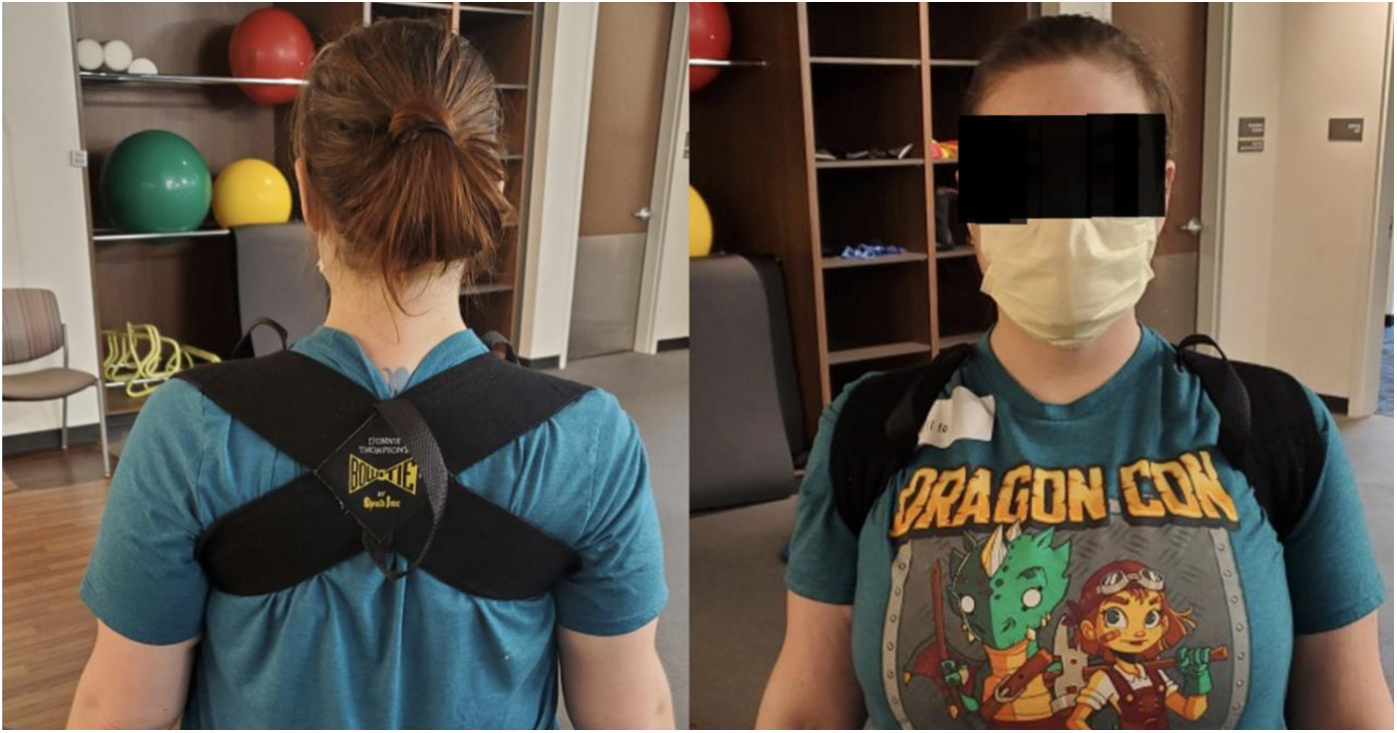
Figure of Eight Brace. The brace combats resting scapular protraction by exerting a constant posterior force on the scapula. This augments postural retraining and relieves compression in the retropecotalis minor space induced by a protracted scapular position.

Initial treatment of PMS is nonoperative, focusing on periscapular muscle stretching and postural retraining. The goal is improving PM length and flexibility while retraining scapular mechanics and scapulohumeral rhythm. To lengthen and stretch PM, the coracoid insertion is moved away from the anterior rib origin.^40^ Specific techniques are depicted in Figures 14 and 15.^19^ Additional exercises to retrain scapular kinematics are also incorporated (Figs. 16 and 17). Orthotic bracing (figure-of-eight) is used to counteract chronic scapular protraction by maintaining shoulders in passive retraction (Fig. 18).

Various stretching techniques for PM lengthening have been described with mixed results.^14^^,^^60^^,^^82^^,^^107^ Borstad et al^14^ compared 3 different techniques and found unilateral self-stretch was superior to supine or sitting manual stretch.^39^ Other studies found not only PM lengthening but also greater scapular upward rotation and posterior scapular tilting after stretching.^60^^,^^107^ In a cohort of 46 young, active patients, Provencher et al^82^ found 40 (87%) responded to stretching and scapular retraining with improved scapular positioning, shoulder function, and pain.

Other studies assessing stretching have equivocal results.^70^^,^^87^ Two studies of home exercise programs involving PM stretching did not show differences in PM length or scapular kinematics but did report decreased symptoms and improved function.^70^^,^^87^

### Surgical management

**Figure 19:**
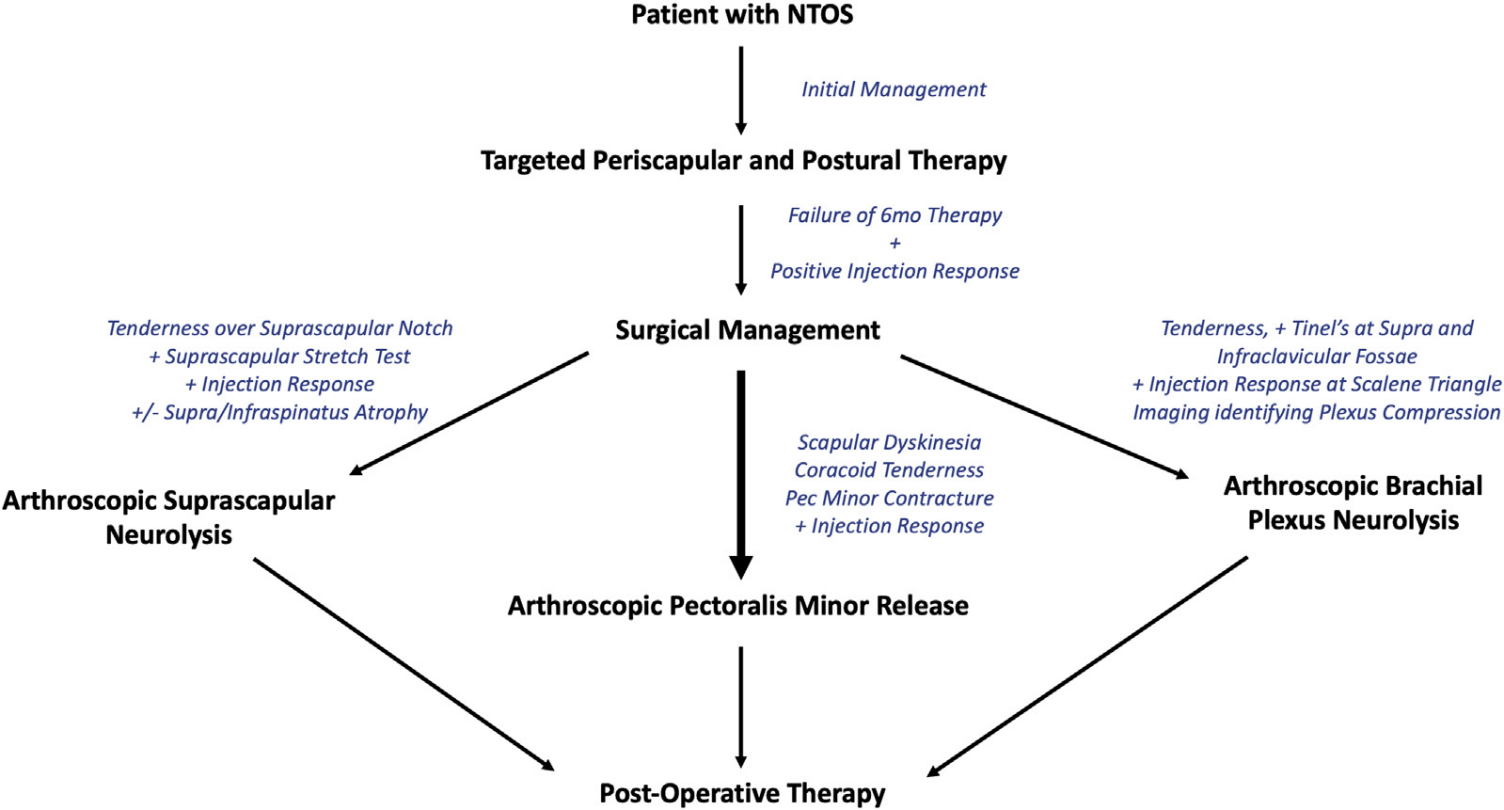
Stepwise treatment for a patient with neurogenic thoracic outlet syndrome. Initial management is conservative, and surgical treatment is reserved for patients failing to improve with therapy and who also demonstrate a positive response (improvement, even transient) to image-guided anesthetic injection. These patients are treated with arthroscopic surgery, often entailing pectoralis minor release. Concomitant suprascapular neurolysis and/or brachial plexus neurolysis are performed based on preoperative work-up as detailed above.

For patients with continued symptoms despite scapular-focused and extensive (6 months) nonoperative treatment, surgical management for PM release is appropriate (Fig. 19). For over 50 years, procedures such as the Latarjet and open brachial plexus exploration incorporated release of PM off the coracoid without adverse consequence.^4^^,^^73^ Recently, isolated PM release for recalcitrant PMS causing NTOS has been advocated. Surgical release of PM has been described via open^72^^,^^82^^,^^94^^,^^103^ and arthroscopic techniques^40^^,^^54^^,^^55^ with initial promising results, although most studies only report short-term outcomes.

Prior to detailing specific techniques, distinction must be made regarding overall surgical management of NTOS vs. VTOS (Table I). As PM tightness and ensuing scapular dyskinesia are the prime agents causing NTOS, it follows that surgical algorithm for NTOS primarily addresses the PM. Secondary effects of PMS, such as suprascapular neuropathy and proximal brachial plexus compression in long-standing cases, are addressed simultaneously pending diagnostic workup. If SSN entrapment is found, release at the suprascapular notch is performed simultaneously. Similarly, if proximal plexus compression is discovered preoperatively, brachial plexus neurolysis is undertaken.

Surgical management of VTOS follows a similar anatomic framework. Primary cause of VTOS is dynamic compression of subclavian vessels between the clavicle and first rib during scalene muscle contraction.^28^^,^^43^^,^^48^ Therefore, first rib resection targets the principal pathoanatomy creating VTOS.^101^ Additional agents precipitating VTOS include anomalous structures, scalene hypertrophy, or pathology intrinsic to subclavian vessels themselves.^23^ Akin to addressing secondary factors in NTOS, these supplementary etiologies in VTOS are addressed per preoperative workup with scalenectomy, resection of anomalous anatomy, or vascular reconstruction.^64^^,^^93^ Outcomes of first rib resection are detailed in multiple studies, varying based on underlying diagnosis,^10^^,^^30^^,^^34^ surgical approach,^1^^,^^10^^,^^30^ and robotic assistance.^51^ A systematic review analyzing surgical treatment of TOS by Peek et al^81^ found first rib resection with or without scalenectomy yielded good or excellent results in 90% of VTOS patients. However, in NTOS patients, this resolved symptoms in a less-consistent 58%-89% range. Furthermore, the largest included study by Vemuri et al^103^ compared isolated PM release vs. PM release combined with first rib resection and scalenectomy. Isolated PM release demonstrated significantly improved DASH score at 3 months compared with the combined group (26.6 vs. 41.5). Peek et al^81^ reported complication rates stemming from first rib resection with or without scalenectomy of up to 40%, including pneumothorax, hematoma requiring evacuation, neurologic injury, and infection. They concluded the greatest challenge in treating TOS is the diagnosis itself, particularly of NTOS, given that no standard algorithm exists. Another systematic review by Yin et al^109^ evaluated outcomes in TOS patients with and without first rib resection. They found a mean success rate of 76% and 77% for transaxillary and supraclavicular first rib resection, respectively, and 85% for supraclavicular release without first rib resection. They found mean success rate of 76% and 77% for transaxillary and supraclavicular first rib resection, respectively, and 85% for supraclavicular release without first rib resection. Avoiding first rib resection had the highest likelihood of achieving complete symptom relief. Finally, complication rates were approximately twice as high for transaxillary and supraclavicular first rib resection (22.5% and 25.9%, respectively) compared to supraclavicular release without first rib resection (12.6%). Majority of these complications, such as pneumothorax and neurologic injury, did not have permanent sequelae, with rates of less than 1% for permanent plexus injury or death in the rib resection group. Permanent complications did not occur in the group without rib resection. These data suggest that first rib resection offers viable treatment for VTOS but is less reliable and effective for NTOS. As detailed in the vascular surgery literature, Ambrad-Chalea et al^5^ recognized PMS as a causative factor in patients with residual symptoms after thoracic outlet decompression involving first rib resection and scalenectomy. Sanders et al^94^ further utilized open transaxillary approach for PM release in 100 patients: 52 with PMS alone and 48 with PMS and additional proximal compression diagnosed using PM and scalene blocks. In the isolated PMS group, they noted 90% good or excellent result compared to 35% in the combined (PMS and scalene compression) group. They noted failure with isolated PM decompression of 8% in the PMS group compared to 46% with both sites involved. Three patients had early wound infections and 15% reported paresthesia on the undersurface of their arm related to injury of the intercostal brachial cutaneous nerve. Vemuri et al^103^ performed PM tenotomy in 52 patients with isolated PMS, diagnosed via examination of predominantly infraclavicular tenderness.^68^ They noted 75% of patients exhibited improvement in symptoms and function at 3 months.

In the orthopedic surgery literature, McIntyre^72^ in 1975 described open release of PM in 10 patients. All patients reported relief of radiating arm pain and return to work within 6 weeks. Three decades later, Provencher et al^82^ evaluated the surgical release of PM in 6 patients who failed conservative management, via mini-open deltopectoral approach. There was significant improvement in pain and shoulder scores, as well as improved scapular motion in all patients. No surgical complications were noted, and all patients returned to full activity.

**Figure 20:**
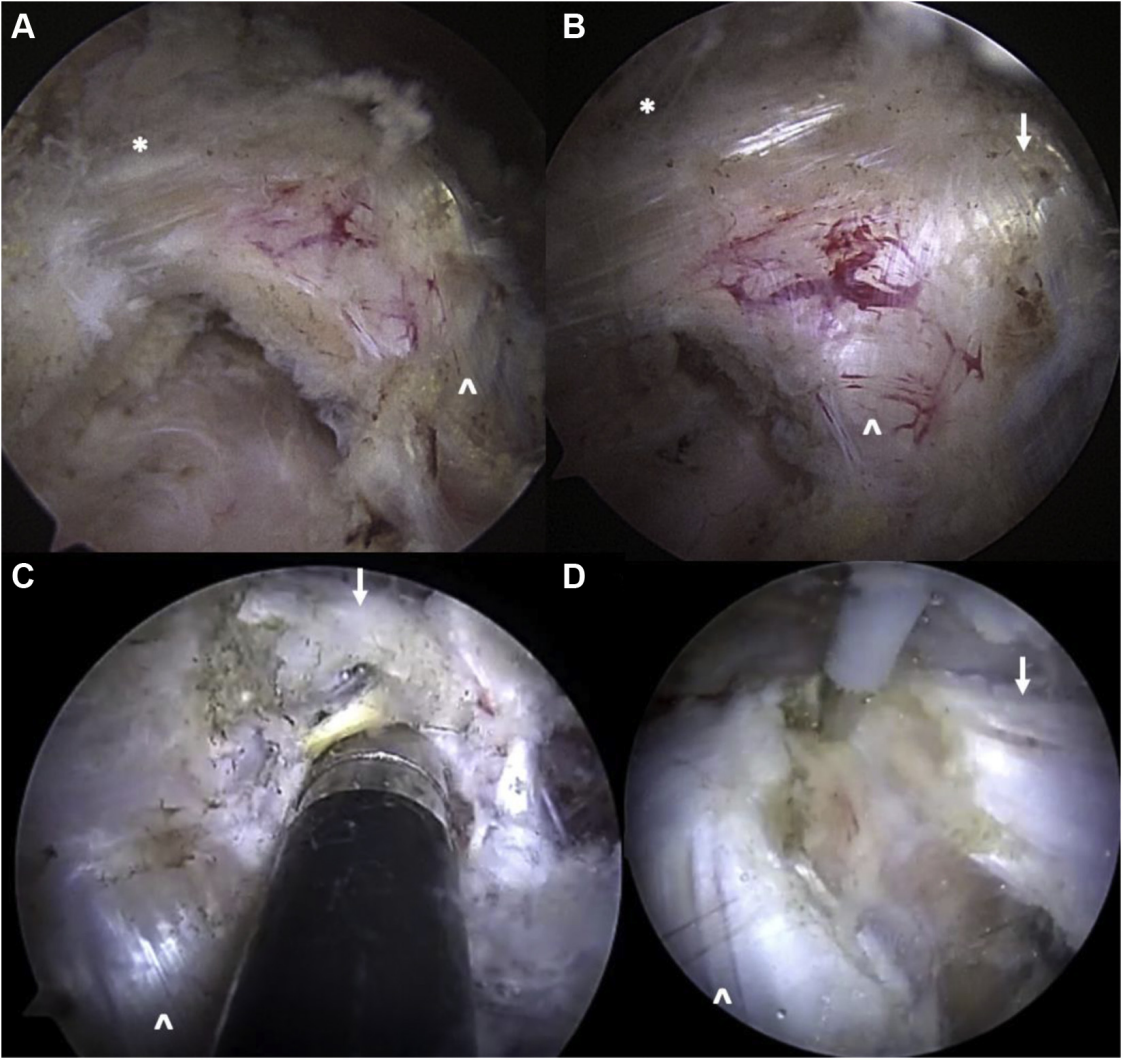
Arthroscopic pectoralis minor release of the right shoulder. (**A**) View from anterolateral portal with standard 30° arthroscope, demonstrating the coracoacromial ligament (∗) and conjoint tendon (**ˆ**). (**B**) View from same portal with 70° arthroscope, demonstrating the classic “T” appearance of the coracoacromial ligament (∗), conjoint tendon (**ˆ**), and pectoralis minor (*downward white arrow*) converging on the coracoid process. (**C**) Release of the pectoralis minor tendon insertion (*downward white arrow*) off the medial coracoid using electrocautery. Conjoint tendon is also seen in this view (**ˆ**). (**D**) Continued release off the coracoid with inferior and medial retraction of the pectoralis minor tendon (*downward white arrow*). This retraction is routinely noted in patients with pectoralis minor syndrome.

**Figure 21:**
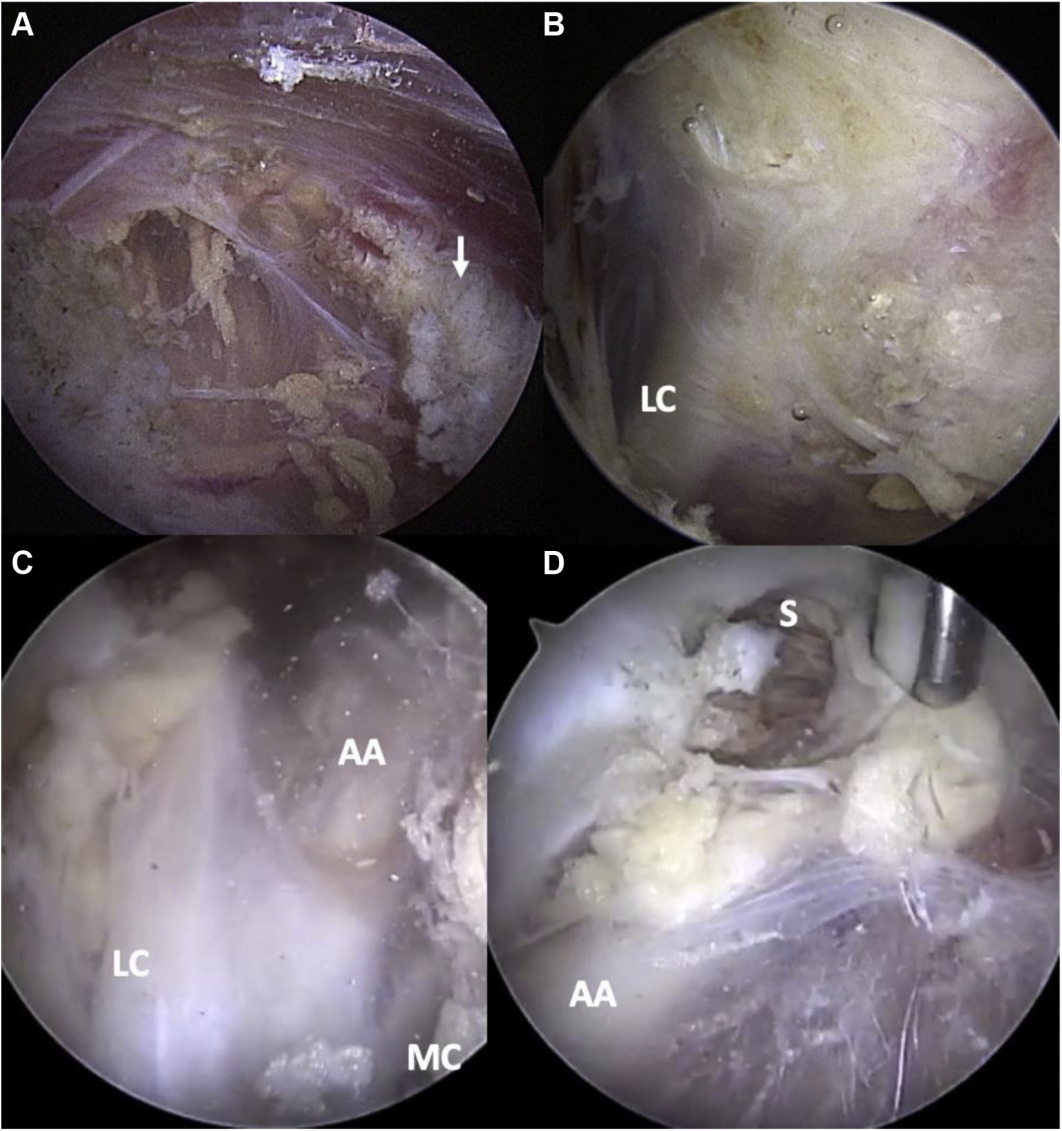
Brachial plexus arthroscopic neurolysis following pectoralis minor release (the same patient as Fig. 20). (**A**) Pectoralis minor completely released (*downward white arrow*) with classic inferomedial retraction. Retropectoralis minor space is now open. (**B**) Release of adhesions and areolar tissue in the retropectoralis minor space uncovers the lateral cord of the brachial plexus. (**C**) Continued proximal release uncovers the axillary artery. Medial cord is partially visualized at the *Bottom Right* portion of the photo. (**D**) Further proximal release presents the subclavius muscle on the inferior surface of the clavicle. This is released, completing the infraclavicular release. *AA*, axillary artery; *LC*, lateral cord; *MC*, medial cord; *S*, subclavius muscle.

A recent innovation in shoulder arthroscopy is arthroscopic PM release (Fig. 20). Though technically challenging, Lafosse et al^56^ have shown this arthroscopic/endoscopic PM release and concomitant brachial plexus neurolysis are possible, reproducible, and safe in skilled hands. Their surgical technique allows for arthroscopic access to the subcoracoid, subdeltoid, and retropectoralis minor space for PM tenotomy off the coracoid. Further proximomedial advancement permits brachial plexus neurolysis and visualization of axillary vessels (Fig. 21).^55^ Their group performed arthroscopic brachial plexus neurolysis and PM release in 36 patients, with resolution of symptoms and no postoperative complications.^54^ In another series of arthroscopic PM release, the senior author was involved in a multicenter study examining outcomes of arthroscopic PM release in 21 patients with a mean 19-month follow-up. Overall, 20 of 21 (95%) had substantial relief of their symptoms with no complications at the time of the latest follow-up (unpublished data).

**Figure 22:**
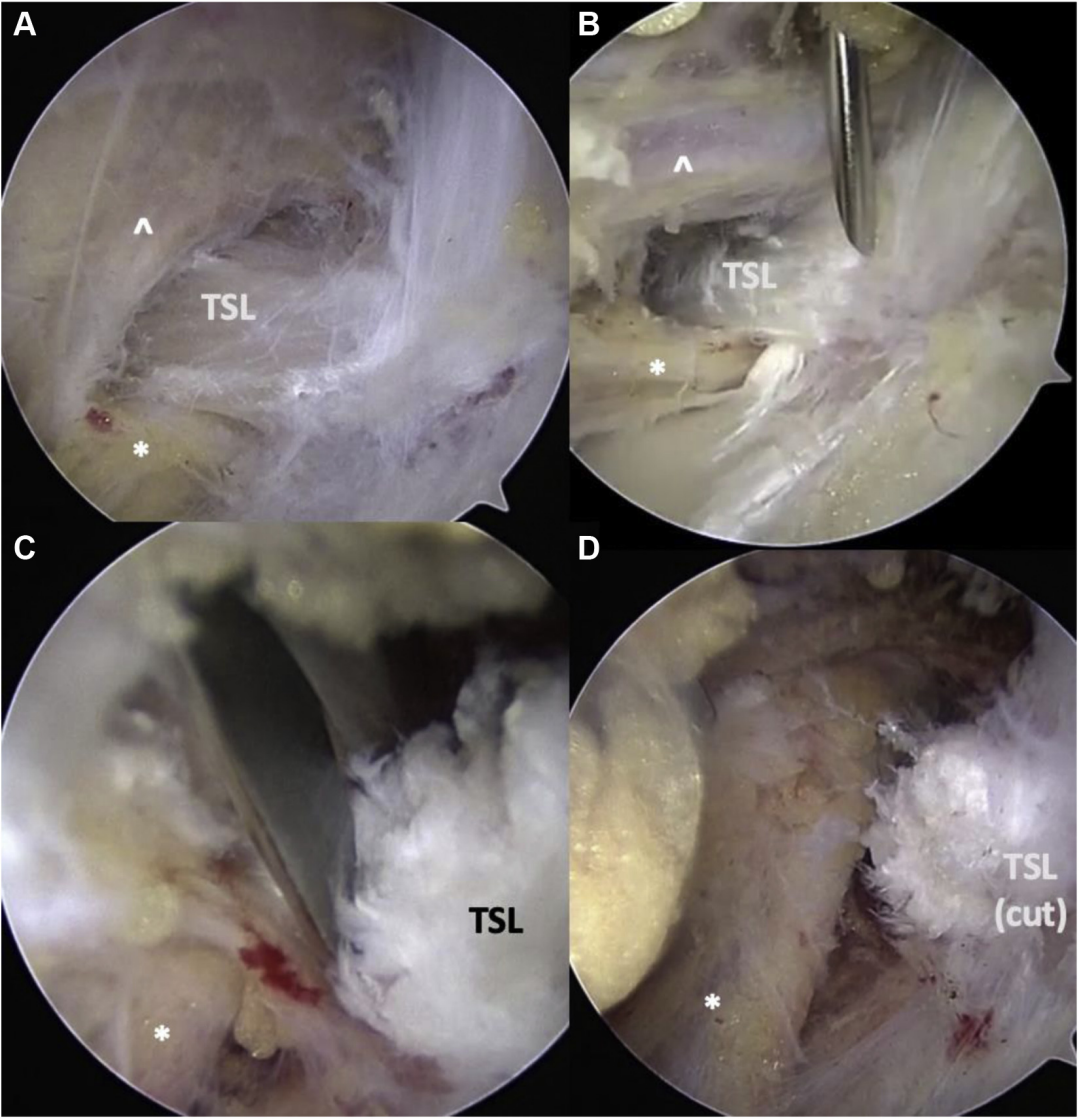
Arthroscopic suprascapular nerve release of the right shoulder (the same patient as Figs. 20 and 21). (**A**) View from subacromial space via lateral portal and 30^o^ arthroscope, progressing medially following the CA ligament and releasing along the anterior border of the supraspinatus muscle until the transverse scapular ligament is encountered posterior to the coracoid. (**B**) Needle localization creating superomedial working portal. (**C**) Arthroscopic scissors introduced through this working portal, releasing transverse scapular ligament. The suprascapular nerve is safely visualized inferior to the ligament. The suprascapular artery runs anterior to posterior over the ligament and is displaced posterior-medial to the scissors to ensure it remains protected. (**D**) Released suprascapular nerve. *CA*, carotid artery; *TSL*, transverse scapular ligament; ∗, indicates suprascapular nerve; **ˆ**, indicates suprascapular artery.

In patients with both SSN entrapment and PMS (stages 3 and 4), arthroscopic approach involves complete infraclavicular thoracic outlet release. The SSN release is performed first, followed by PM release, and finally brachial plexus neurolysis proximally to the level of the subclavius muscle. After arthroscopic SSN decompression via release of the transverse scapular ligament (Fig. 22), further medial advancement often displays fibrous bands and adhesions that are released toward the subclavius muscle at the inferior surface of the clavicle. Attention is then turned to the PM release, as depicted in Figure 20. After this is completed, continued superomedial brachial plexus neurolysis is performed by following the plexus cords proximally until the subclavius muscle is encountered on the inferior surface of the clavicle. The subclavius is fully débrided (Fig. 21), along with any residual fibrous bands or adhesions. Once this is complete, the entire infraclavicular thoracic outlet is decompressed.

Postoperative protocol is detailed in Table III. Early range of motion and targeted stretching program begins under therapist guidance, along with use of figure-of-eight brace to reverse protracted resting scapular posture. These protocols involve comprehensive PM stretching, postural retraining, and scapulohumeral rhythm retraining.^14^^,^^19^^,^^60^^,^^82^^,^^107^ Aggressive periscapular muscle strengthening is initiated between 4 and 6 weeks, with most patients returning to overhead activity, including sports, by 3 to 4 months postoperatively. During rehabilitation, strict attention to core, hip, and lower extremity strengthening and coordination is emphasized.^40^^,^^82^

**Table 3:** Postoperative protocol after arthroscopic pectoralis minor release.

Weeks 0-2	Phase 1: Immobilization	- Simple sling immediately postoperatively - Transition to Figure-of-Eight brace at the first postoperative visit - Passive and active elbow, wrist, and hand motion
Weeks 2-6	Phase 2: Range of motion and scapula retraining	- Progress from passive to active assisted to active shoulder motion - Periscapular strengthening, retrain scapular kinematics, and pectoralis minor stretching - Continue using Figure-of-Eight brace - Pool therapy encouraged
Weeks 6-12	Phase 3: Strengthening	- Full active/passive shoulder motion - More aggressive strengthening with progression to eccentric strengthening - Continue postural retraining and scapulohumeral rhythm kinematics, continue use of Figure-of-Eight brace
Weeks 12-16	Phase 4: Sport and activity specific	- Continue Phase 3 therapy - Wean use of Figure-of-Eight brace - Gradual return to sport and activity

## Conclusion

PMS and resultant NTOS are challenging entities to recognize, diagnose, and treat. Sound understanding of scapulothoracic mechanics and thoracic outlet anatomy are essential for all providers who treat these patients. Diagnostic workup must be thorough to rule out various etiologies mimicking NTOS. Ultrasound-guided injections are a mainstay of diagnosis and recommended for patients with suspected NTOS prior to invasive treatment. A majority of patients improve with therapy targeted at correcting scapular dyskinesia and stretching of the pectoralis minor. In recalcitrant cases, open or arthroscopic pectoralis minor release yields high rates of success and can be combined with SSN and brachial plexus neurolysis.

## Disclaimers:

Funding: No funding was disclosed by the authors.

Conflicts of interest: Michael B. Gottschalk reports being an Acumed consultant and that he recieved institutional support from Arthrex, Acumed, Skeletal Dynamics, Stryker PI research funding. None are relevant to this manuscript. Eric R. Wagner reports receiving consulting fees from Stryker, Wright Medical, Biomet, Acumed, and Osteoremedies and research support from Arthrex, Konica Minolta, Arthrex, and DJO. None are relevant to this manuscript. The other authors, their immediate families, and any research foundation with which they are affiliated have not received any financial payments or other benefits from any commercial entity related to the subject of this article.
